# The ubiquitin-specific protease USP36 SUMOylates EXOSC10 and promotes the nucleolar RNA exosome function in rRNA processing

**DOI:** 10.1093/nar/gkad140

**Published:** 2023-03-13

**Authors:** Yingxiao Chen, Yanping Li, Roselyn S Dai, Jonathan C Savage, Ujwal Shinde, John Klimek, Larry L David, Emma A Young, Markus Hafner, Rosalie C Sears, Xiao-Xin Sun, Mu-Shui Dai

**Affiliations:** Department of Molecular & Medical Genetics, 3181 SW Sam Jackson Park Road, Portland, OR 97239, USA; Department of Molecular & Medical Genetics, 3181 SW Sam Jackson Park Road, Portland, OR 97239, USA; Department of Molecular & Medical Genetics, 3181 SW Sam Jackson Park Road, Portland, OR 97239, USA; Department of Chemical Physiology & Biochemistry, School of Medicine, 3181 SW Sam Jackson Park Road, Portland, OR 97239, USA; Department of Chemical Physiology & Biochemistry, School of Medicine, 3181 SW Sam Jackson Park Road, Portland, OR 97239, USA; OHSU Proteomics Shared Resource, Oregon Health & Science University, 3181 SW Sam Jackson Park Road, Portland, OR 97239, USA; Department of Chemical Physiology & Biochemistry, School of Medicine, 3181 SW Sam Jackson Park Road, Portland, OR 97239, USA; OHSU Proteomics Shared Resource, Oregon Health & Science University, 3181 SW Sam Jackson Park Road, Portland, OR 97239, USA; Laboratory of Muscle Stem Cells and Gene Regulation, National Institute for Arthritis and Musculoskeletal and Skin Disease, National Institutes of Health, Bethesda, MD 20892, USA; Laboratory of Muscle Stem Cells and Gene Regulation, National Institute for Arthritis and Musculoskeletal and Skin Disease, National Institutes of Health, Bethesda, MD 20892, USA; Department of Molecular & Medical Genetics, 3181 SW Sam Jackson Park Road, Portland, OR 97239, USA; Department of Molecular & Medical Genetics, 3181 SW Sam Jackson Park Road, Portland, OR 97239, USA; Department of Molecular & Medical Genetics, 3181 SW Sam Jackson Park Road, Portland, OR 97239, USA

## Abstract

The RNA exosome is an essential 3′ to 5′ exoribonuclease complex that mediates degradation, processing and quality control of virtually all eukaryotic RNAs. The nucleolar RNA exosome, consisting of a nine-subunit core and a distributive 3′ to 5′ exonuclease EXOSC10, plays a critical role in processing and degrading nucleolar RNAs, including pre-rRNA. However, how the RNA exosome is regulated in the nucleolus is poorly understood. Here, we report that the nucleolar ubiquitin-specific protease USP36 is a novel regulator of the nucleolar RNA exosome. USP36 binds to the RNA exosome through direct interaction with EXOSC10 in the nucleolus. Interestingly, USP36 does not significantly regulate the levels of EXOSC10 and other tested exosome subunits. Instead, it mediates EXOSC10 SUMOylation at lysine (K) 583. Mutating K583 impaired the binding of EXOSC10 to pre-rRNAs, and the K583R mutant failed to rescue the defects in rRNA processing and cell growth inhibition caused by knockdown of endogenous EXOSC10. Furthermore, EXOSC10 SUMOylation is markedly reduced in cells in response to perturbation of ribosomal biogenesis. Together, these results suggest that USP36 acts as a SUMO ligase to promote EXOSC10 SUMOylation critical for the RNA exosome function in ribosome biogenesis.

## INTRODUCTION

Ribosome biogenesis is a multi-step and highly orchestrated cellular process for making the ribosome, including rRNA synthesis and processing, ribosome subunit assembly in the nucleolus and subsequent transport into the cytoplasm (1,2). It requires the assistance of >200 ribosome biogenesis accessory factors (1), including the nucleolar RNA exosome (3–5). The eukaryotic RNA exosome is a multi-subunit protein complex that catalyzes 3′ to 5′ processing or degradation of RNA substrates (3–5). Its function includes the processing and degradation of virtually all RNAs including rRNA, tRNAs, small nuclear RNAs (snRNAs) and small nucleolar RNAs (snoRNAs) in the nucleus, mRNA turnover in the cytoplasm and the surveillance of aberrant RNAs throughout the cell (3–9). The complex contains a barrel-shaped nine-subunit catalytically inactive core (called Exo9) consisting of a three-subunit S1-KH ‘cap’ and a hexameric PH ‘ring’ (Supplementary Figure S1A). The ‘cap’ is composed of the S1 and KH RNA-binding domain containing proteins Csl4, ribosomal RNA processing protein 4 (Rrp4) and Rrp40. The PH ‘ring’ contains six RNase PH-like domain-containing proteins Rrp41, Rrp42, Rrp43, Rrp46, Mtr3 and Rrp45 (3–5,10–12). The core complex associates with the processive 3′ to 5′ exo- and endoribonuclease Dis3 at the bottom of the PH-protein barrel and/or the distributive 3′ to 5′ exonuclease Rrp6 at the cap side, forming the exosome complexes Exo10^Dis3^, Exo10^Rrp6^ and Exo11^Dis3+Rrp6^, respectively (3–5,10–12). In yeast, Exo10^Dis3^ exists in the cytoplasm, whereas the nuclear RNA exosome is Exo11^Dis3+Rrp6^. In human, Dis3 is excluded from the nucleolus and the Dis3 homolog Dis3L associates with the exosome core in the cytoplasm, whereas the nucleolar RNA exosome is Exo10^hRrp6^ (3–5,13,14) (Supplementary Figure S1B).

As a critical player in ribosome biogenesis, human Rrp6 (also known as exosome component 10, EXOSC10) has been shown to function in the turnover of the 5′-external transcribed spacer (ETS) (15) and the processing of internal transcribed spacer (ITS) 1 (16) and ITS2 (7,8,16,17) of the precursor rRNA (pre-rRNA), and mature snoRNA turnover (17). A recent study showed that the main direct targets of human EXOSC10 include 3′-extended 5.8S rRNA and 3′-extended snoRNAs, indicating that EXOSC10 mainly functions in 5.8S rRNA maturation and the final steps of snoRNA processing (17). In addition, a number of cofactor proteins are required for proper exosome function (3–5). For example, the DExH helicase Mtr4 and superkiller 2 (Ski2) are required for RNA degradation in the nucleus and the cytoplasm, respectively (18,19). Mtr4 interacts with a non-canonical poly(A) polymerase (Trf4 or Trf5 in yeast and PAPD5 in human) and a Zn-knuckle RNA-binding protein arginine methyltransferase-interacting RING finger 1 (Air1) or Air2 (ZCCHC7 in human) to form the Trf4/5-Air1/2-Mtr4 polyadenylation (TRAMP) complex (20–23). In human, the TRAMP complex consists of MTR4 (also called Skiv2l2), PAPD5 and ZCCHC7, and is exclusively present in the nucleolus and involved in rRNA processing (4,5,24). Thus, rRNA processing involves complex exosome proteins and their cofactors. However, how these processes are regulated in the nucleolus is largely unknown.

The nucleolar deubiquitinating enzyme (DUB) USP36 plays a critical role in ribosome biogenesis (25–29) by deubiquitinating and stabilizing several nucleolar proteins, including nucleophosmin (NPM) (26), fibrillarin (FBL) (26), the RNA helicase DHX33 (27) and the RNA polymerase I (Pol I) subunit Rpa190 (28). USP36 also deubiquitinates and stabilizes c-Myc, a master regulator of ribosome biogenesis (30,31), and controls c-Myc degradation in the nucleolus (29,32). Interestingly, we recently identified that USP36 also acts as a novel SUMO ligase that promotes small nucleolar ribonucleoprotein (snoRNP) protein group SUMOylation (33). SUMOylation is a post-translational modification of proteins by small ubiquitin-like modifiers (SUMOs) and plays critical roles in regulating protein localization, trafficking, stability and activity (34,35) by interfering with protein–protein interactions through steric hindrance (36) or competing with other lysine-directed modifications such as acetylation or ubiquitination (37). Consequently, SUMOylation regulates diverse cellular processes, including transcription, chromatin dynamics, DNA replication and repair, RNA splicing and processing, cell cycle control and ribosome biogenesis (34,35,38–40). A number of nucleolar ribosome biogenesis-associated proteins are modified by SUMO, such as NPM (41,42), nucleolin (43), Las1L (44,45), Pelp1 (46) and snoRNP complex components Nop58, Nop56, Nhp2 and DKC1 (33,47,48). Proteomic studies also found that ribosome biogenesis-related proteins are one of the major groups of SUMOylated proteins (49–53). Thus, USP36 acting as a SUMO E3 reveals an additional mechanism underlying its crucial role in ribosome biogenesis (33).

In this study, we found that USP36 associates with the RNA exosome through interaction with its catalytic subunit EXOSC10. USP36 does not significantly affect the levels of EXOSC10 and other tested exosome subunits. Instead, it mainly acts as a SUMO ligase to mediate the SUMOylation of EXOSC10 at lysine (Lys, K) 583. Mutating K583 impaired the binding of EXOSC10 to pre-rRNAs, and the K583R mutant failed to rescue the defects in rRNA processing, protein translation and cell growth caused by knockdown of endogenous EXOSC10. These results suggest that USP36 acts as a SUMO ligase to SUMOylate EXOSC10 and promote the nucleolar RNA exosome function in ribosome biogenesis and cell growth.

## MATERIALS AND METHODS

### Cell culture, plasmids and recombinant proteins

Human H1299, HeLa, 293 and U2OS cells were cultured in Dulbecco's modified Eagle's medium (DMEM) supplemented with 10% (v/v) fetal bovine serum (FBS), 50 U/ml penicillin and 0.1 mg/ml streptomycin at 37°C in a 5% CO_2_ humidified atmosphere as previously described (29,33,54).

Flag-tagged full-length USP36 [wild type (WT) and the C131A mutant] and deletion mutants were previously described (29,33). V5-tagged EXOSC10 was obtained from Addgene (plasmid No: 64916). V5-EXOSC10^K168R^ and V5-EXOSC10^K583R^ mutants were generated by site-directed mutagenesis using the QuikChange Kit (Agilent). EXOSC10 cDNAs (WT and the K583R mutant) were also cloned by polymerase chain reaction (PCR) into the pcDNA3-2Flag vector to generate Flag-EXOSC10 and Flag-EXOSC10^K583R^ plasmids. All flag-tagged EXOSC10 deletion mutants were constructed by inserting PCR products into pcDNA3-2Flag vector. His-tagged SUMO1, SUMO2 and ubiquitin (Ub) plasmids have previously been described (33,55). The EXOSC10 cDNAs were also cloned into the pcDNA4-TO vector (Life Technologies) to generate pcDNA4-TO-EXOSC10 and pcDNA4-TO-EXOSC10^K583R^ plasmids. These plasmids were then used to construct the EXOSC10_siRNA_Res plasmids by mutagenesis to generate tetracycline (Tet)-inducible expression of siRNA-resistant EXOSC10 (WT and the K583R mutant), in which the EXOSC10 small interfering RNA (siRNA) targeting sequence (siRNA-1) 5′-GGATGAGTCCTACCTTGAA-3′ was mutated to 5′-GGACGAATCTTATTTGGAA-3′. EXOSC10 cDNA was subcloned into the pGEX.4T.1 vector (GE Healthcare) to express the glutathione *S*-transferase (GST)–EXOSC10 fusion protein. USP36 cDNA was inserted into the pET30a vector to generate pET30a-His-USP36 for the expression of recombinant His-USP36 protein in bacteria. All the plasmids were confirmed by sequencing.

Recombinant proteins were expressed in *Escherichia coli* (BL21) by induction with isopropyl-β-d-1-thiogalactopyranoside (IPTG) and purified using glutathione agarose for GST fusion proteins and Ni^2+^-NTA agarose beads for His-tagged proteins. The expression and purification were described previously (29,33,54). Recombinant SUMO E1 (SAE1/SAE2), Ubc9 and SUMO1 proteins were purchased from Boston Biochem.

### Antibodies and reagents

Anti-Flag (M2, F3165, Sigma), anti-V5 (R960-25, Life Technologies), anti-EXOSC10 (sc-374595, Santa Cruz Biotechnology), anti-EXOSC10 (A303-98A,Bethyl Laboratory), anti-hRrp40 (EXOSC3) (sc-166568, Santa Cruz Biotechnology), anti-EXOSC3 (sc-98776, Santa Cruz Biotechnology), anti-hRrp41(EXOSC4) (sc-166772, Santa Cruz Biotechnology), anti-hRrp4 (EXOSC2) (A303-886A, Bethyl Laboratory), anti-MTR4 (A300-614A, Bethyl Laboratory), anti-Dis3 (A303-765A, Bethyl Laboratory), anti-Nop58 (A302-719A, Bethyl Laboratory), anti-Las1L (A304-438A, Bethyl Laboratory), anti-puromycin (clone 12D10, MABE343, EMD Millipore), anti-Ub (sc-9133, Santa Cruz Biotechnology), anti-c-Myc (ab32072, abcam), anti-SP1 (07–645, EMD Millipore), anti-Digoxigenin-AP (11093274910, Roche), anti-USP36 (14783–1-AP, Proteintech) and anti-RPL30 (sc-98106, Santa Cruz Biotechnology) antibodies were purchased. Anti-RPL5, anti-RPL11 and anti-RPS27a were described previously (56–58). Rabbit polyclonal anti-SUMO1 and anti-SUMO2/3 antibodies were provided by Dr Yoshiaki Azuma (University of Kansas). Rabbit anti-USP36 serum was provided by Dr Masayuki Komada (Tokyo Institute of Technology, Japan) (26,29). Puromycin (Life Technologies), doxycycline (Dox; Sigma), actinomycin D (Act D; Sigma), CX-5461 (Sigma), RNase A (Thermo Scientific) and RNase T1 (Thermo Scientific) were purchased.

### Generation of Tet-inducible EXOSC10 expression cell lines

To generate Tet-inducible expression of EXOSC10, HeLa cells were first transfected with pcDNA6-TR (Life Technologies) followed by selection in 5 μg/ml blasticidin-containing medium to establish HeLa cells stably expressing TR (HeLa-TR). HeLa-TR cells were then transfected with pcDNA4-TO-EXOSC10^si1-res^ (WT and the K583R mutant) and selected in medium containing 5 μg/ml blasticidin and 100 μg/ml zeocin for up to 2 weeks. Single colonies were isolated, expanded and screened by immunoblot analysis for Dox (2 μg/ml)-induced expression of EXOSC10 using anti-EXOSC10 antibody. All the cells were cultured in DMEM supplemented with 10% tetracycline system-approved FBS.

### Transfection, immunoblot (IB) and co-immunoprecipitation (co-IP) analyses

Cells were transfected with plasmids using Lipofectamine 2000 (Life Technologies) or TransIT®-LT1 reagents (Mirus Bio Corporation) following the manufacturers’ protocol. Cells were harvested at 36–48 h post-transfection and lysed in NP-40 lysis buffer consisting of 50 mM Tris–HCl (pH 8.0), 0.5% Nonidet P-40, 1 mM EDTA, 150 mM NaCl, 1 mM phenylmethylsulfonyl fluoride (PMSF), 1 mM dithiothreitol (DTT), 1 μg/ml pepstatin A and 1 mM leupeptin. Equal amounts of total protein were used for IB analysis. Co-IP was conducted as described previously (29). Bound proteins were detected by IB using antibodies as indicated in the figure legends.

### Affinity purification of human USP36-associated protein complexes and mass spectrometry analysis

293 cells stably expressing control or Flag-USP36 were established by transfecting with control pcDNA3-Flag and Flag-USP36 plasmids, respectively, followed by selection in medium containing 0.5 mg/ml neomycin (G418) for single clones. The cells were lysed in NP-40 lysis buffer supplemented with protease inhibitors at 4°C for 1 h followed by centrifugation. A 20 mg aliquot of the cleared cell lysates from either control or Flag-USP36-expressing cells was incubated with 0.1 ml of anti-Flag (M2) agarose beads at 4°C for 4 h. The beads were washed four times in lysis buffer containing protease inhibitors. The bead-bound proteins were eluted in 0.2 ml of Tris-buffered saline (TBS; 50 mM Tris–HCl, 150 mM NaCl, pH 7.4) containing 0.1 mg/ml Flag peptides. Eluted proteins were run into a sodium dodecylsulfate–polyacrylamide gel electrophorsis (SDS–PAGE) gel for 6 min, the gel was stained, and protein bands at the top of the gel were excised, cut into 1 mm pieces, reduced/alkylated and digested with trypsin for 1 h at 50°C in the presence of ProteaseMax™ detergent using the method recommended by the manufacturer (Promega). Recovered peptides were then dried by vacuum centrifugation, dissolved in 5% formic acid and loaded onto an Acclaim PepMap 0.1 × 20 mm NanoViper C18 peptide trap (Thermo Scientific) for 5 min at 10 μl/min in a 2% acetonitrile (ACN), 0.1% formic acid mobile phase. Peptides were then separated using a PepMap RSLC C18, 2 μm particle, 75 μm × 50 cm EasySpray column using a 7.5–30% ACN gradient over 90 min in a mobile phase containing 0.1% formic acid and a 300 nl/min flow rate provided by a Dionex NCS-3500RS UltiMate RSLC nano UPLC (Thermo Scientific). Tandem mass spectrometry (MS/MS) data were collected using an Orbitrap Fusion mass spectrometer (Thermo Scientific) configured for data-dependent analysis. MS1 scan resolution was set to 120 000 (at *m/z* 200) and the MS1 automatic gain control (AGC) target was 200 000 with a maximum injection time of 50 ms. Mass range was set at 400–1500. MS1 data were acquired in profile mode using positive polarity, a MIPS filter on with relaxed conditions and charge states from +2 to +7 accepted. The linear ion trap AGC target value for fragment spectra was set at 10 000, intensity threshold was 5000 and a rapid scan rate was used. Quadrupole isolation width was set at 1.6 *m/z*. Normalized higher-energy collisional dissociation (HCD) was set at 35%. Dynamic exclusion was set to ±10 ppm with a duration of 30 s. The program Comet (v. 2016.01, rev. 3) was used to search MS2 Spectra against a June 2019 version of a UniProt FASTA protein database containing 20 960 canonical *Homo sapiens* sequences with the addition of the human USP36 sequence, and 179 common contaminant sequences. To estimate error rates, sequence-reversed forms of all proteins were concatenated to the FASTA file. The database processing was performed with Python scripts available at https://github.com/pwilmart/fasta_utilities.git and Comet results processing used the PAW pipeline from https://github.com/pwilmart/PAW_pipeline.git. Comet searches for all samples were performed with trypsin enzyme specificity. Monoisotopic parent ion mass tolerance was 1.25 Da. Monoisotopic fragment ion mass tolerance was 1.0005 Da. A static modification of +113.084 Da was added to all cysteine residues and a variable modification of +79.9663 Da to serine, threonine and tyrosine residues. Comet scores were combined into linear discriminant function scores, and discriminant score histograms were created separately for each peptide charge state (2+, 3+ and 4+). Separate histograms were created for matches to forward sequences and for matches to reversed sequences for all peptides of seven amino acids or longer. The score histograms for reversed matches were used to estimate peptide false discovery rates (FDRs) and to set score thresholds for each peptide class. After removal of contaminants, this resulted in the identification of ∼953 proteins with at least two unique peptides per protein and an estimated protein FDR <0.5%. Estimation of protein abundance differences in the immunoprecipitates from control or Flag-USP36 cell lysates was performed using the numbers of assigned MS/MS spectra (spectral counts) to each peptide from protein across the two samples. Putative interacting proteins were defined by having three or more spectral counts in the Flag-USP36 sample and either 0 spectral counts in the control sample or a ratio of ≥2.5 when dividing the numbers of spectral counts in the Flag-USP36 sample by the spectral counts in the control sample.

### Gene knockdown by RNA interference

Lentiviral vectors encoding short hairpin RNAs (shRNAs) against USP36 and EXOSC10 were purchased (Open Biosystems). The shRNA sequences are 5′-GCGGTCAGTCAGGATGCTATT-3′ (USP36 shRNA) and 5′-ATGAAAGACCTTAACGATGGC-3′ (EXOSC10 shRNA). The plasmids were transfected with VSVG, pLP1 and pLP2 plasmids into 293FT cells using calcium chloride (Promega). The viruses were then used to infect cells in the presence of polybrene (6 μg/ml). The cells were harvested at 72 h post-transduction for IB analysis. For siRNA-mediated knockdown, the 21 nucleotide siRNA duplexes with a 3′-dTdT overhang were synthesized by Dharmacon Inc. (Lafayette, CO, USA). The target sequences for EXOSC10 are 5′-GGATGAGTCCTACCTTGAA-3′ (siRNA-1) and 5′-GCAAGACATGTTTGCACAT-3′ (siRNA-2). The target sequence for USP36 is 5′-TGTCCTGAGTGGAGAGAAT-3′. The control scramble (scr) RNA was described (55). These siRNA duplexes (100 nM) were introduced into cells using Lipofectamine 2000 (Invitrogen) following the manufacturer's protocol.

### Glutathione *S*-transferase fusion protein association assays.

GST fusion protein–protein association assays were conducted as described (29,33,54). Briefly, purified His-USP36 proteins (200 ng) were incubated with glutathione–Sepharose 4B beads (Sigma) containing 200 ng of GST–EXOSC10 and GST alone, respectively, in a final volume of 50 μl of binding buffer containing 50 mM Tris–HCl 7.5, 5 mM MgCl_2_, 100 mM NaCl, 10% glycerol, 0.5 mg/ml bovine serum albumin (BSA), 5 mM β-mercaptoethanol for 45 min at room temperature with gentle agitation. The beads were then washed five times with 500 μl of the binding buffer and bound proteins were analyzed using IB with anti-USP36 antibody.

### *In vivo* ubiquitination and SUMOylation assays

*In vivo* ubiquitination and SUMOylation assays under denaturing conditions were conducted using an Ni^2+^-NTA pulldown (PD) method as previously described (54,55,59). For ubiquitination assay, cells were transfected with His-Ub and the indicated, plasmids and treated with 40 μM MG132 for 6 h before harvesting. The cells were harvested at 48 h after transfection; 20% of the cells were used for direct IB and the rest of cells were subjected to Ni^2+^-NTA PD under denaturing conditions. The bead-bound proteins were analyzed using IB. For SUMOylation assay, cells were transfected with His-SUMO1 and the indicated plasmids followed by Ni^2+^-NTA PD under denaturing conditions similar to that above.

### *In vitro* SUMOylation assay

*In vitro* SUMOylation assays were carried out as described previously (33) in a 20 μl reaction mixture containing SUMO E1 heterodimer (50 nM), Ubc9 (E2, 50 nM), SUMO1 (2 μM), ATP (2.5 mM), Flag-EXOSC10 (20 nM) and Flag-USP36 (0.25 μM) purified from 293T cells in reaction buffer consisting of 20 mM HEPES (pH 7.8), 100 mM NaCl, 5 mM MgCl_2_, 5% glycerol and 1 mM DTT. The reactions were incubated at 30°C for 5 h and then stopped by adding an equal volume of 2× SDS sample buffer, followed by IB.

### Nucleolar fractionation

Nucleolar fractionation was performed as described previously (29). Briefly, freshly harvested cells were washed with phosphate-buffered saline (PBS), resuspended in hypotonic buffer A (10 mM HEPES pH7.8, 10 mM KCl, 1.5 mM MgCl_2_, 0.5 mM DTT) in the presence of PMSF and incubated for 10 min on ice. The cells were homogenized using a B pestle douncer followed by spinning down at 3000 rpm for 5 min at 4°C. The supernatant (cytoplasmic fraction) was supplemented with 1/10 volume of buffer B (0.3 M Tris–HCl pH 7.8, 1.4 M KCl, 30 mM MgCl_2_). The nuclear pellets were washed with buffer A and then resuspended in buffer S1 (0.25 M sucrose, 10 mM MgCl_2_), layered over buffer S2 (0.35 M sucrose, 0.5 mM MgCl_2_) and centrifuged at 1430 *g* for 10 min at 4°C. The pelleted nuclei were resuspended in buffer S2 with PMSF, and sonicated using a microtip probe with the power setting at 50%. The sonicated nuclei were then layered over buffer S3 containing 0.88 M sucrose and 0.5 mM MgCl_2_, and centrifuged at 3000 *g* for 10 min at 4°C. The pellet contained purified nucleoli and the supernatant represented the nucleoplasm.

### Size exclusion chromatography (SEC)

Nuclear extracts were prepared from the above 293 cells stably expressing Flag-USP36 as previously described (59). Briefly, cells were resuspended in hypotonic buffer A, homogenized and centrifuged as above. The resulting nuclear pellets were lysed in buffer C consisting of 20 mM HEPES (pH 7.9), 420 mM NaCl, 0.2 mM EDTA, 1.5 mM MgCl_2_, 0.5 DTT, 25% glycerol with protease inhibitors. A total of 250 μl of the nuclear extracts were loaded onto a Superose 6 10/300 GL column (24 ml, GE) equilibrated with PBS (pH 7.2). The flow rate was 0.03 ml/min, and 56 fractions (300 μl each) were collected automatically. A calibration curve was obtained with standard proteins (Sigma-Aldrich) as run on the same system. Every two fractions were analyzed using IB with antibodies as indicated in the figure legends.

### Immunofluorescence (IF) staining

Cells were fixed and stained with monoclonal anti-Flag, anti-EXOSC10, anti-Nop58 and anti-USP36 antibodies as indicated, followed by staining with Alexa Fluor 488 (green) goat anti-mouse antibody and Alexa Fluor 546 (red) goat anti-rabbit antibody (Invitrogen) as well as 4′,6-diamidino-2-phenylindole (DAPI) for DNA staining. Stained cells were analyzed under a fluorescence microscope (Apotome, Zeiss).

### RNA-immunoprecipitation (RNA-IP) and quantitative reverse transcription–PCR (RT–qPCR)

Cells were lysed in lysis buffer containing 20 mM HEPES pH 7.9, 150 mM NaCl, 1 mM MgCl_2_, 1 mM EGTA, 10% glycerol, 1% Triton-X100, 0.1% sodium deoxycholate in the presence of EDTA-free complete protease inhibitor cocktail (Roche) and 20 U/ml RNase inhibitor (Invitrogen) for 45 min, briefly sonicated and centrifuged at 15 000 *g* for 15 min at 4°C. The supernatants were pre-cleared with protein G beads for 30 min, followed by incubation with anti-Flag (M2)-conjugated beads (Sigma) or control IgG-coated beads for 4 h at 4°C. After washing with lysis buffer four times, the beads were suspended in 100 μl of NT2 buffer (50 mM Tris–HCl pH 7.4, 150 mM NaCl, 1 mM MgCl_2_, 0.05% NP-40) containing 10 U of DNase and incubated at 37°C for 10 min. RNA–protein complexes were eluted with elution buffer (10 mM Tris–HCl pH 8.0, 1 mM EDTA, 1% SDS, 20 U/ml RNase inhibitor) at 65°C for 10 min. The eluates were incubated with Proteinase K (1 mg/ml) at 50°C for 5 h. RNAs were then extracted using Trizol reagent (Invitrogen) and subjected to reverse transcription using the RevertAid RT Reverse transcription Kit (K1691, Thermo Scientific). For EXOSC10 IP, cells were cross-linked with UV light (λ = 254 nm) in an XL-1000 ultraviolet Spectrolinker device at 150 mJ/cm^2^ before harvesting. Quantitative real-time PCR was performed on an ABI StepOne™ real-time PCR system (Applied Biosystems) using SYBR Green Mix (Applied Biosystems) as described previously (55). All reactions were carried out in triplicate. Relative gene expression was calculated using the ΔCτ method following the manufacturer's instruction. The primers used were: 5′-TGCAGGACACATTGATCATCG-3′ and 5′-CGATTGATCGGCAAGCGAC-3′ for the 5.8S–ITS2 junction; 5′-TGAGAAGACGGTCGAACTTG-3′ and 5′-TCCGGGCTCCGTTAATGATC-3′ for the 18S–ITS1 junction; and 5′-ACGAGCGCACGTGTTAGGAC-3′ and 5′-TGCACGTCAGGACCGCTACG-3′ for 28S rRNA.

### Northern blot

Non-radioactive northern blot for rRNA processing was conducted as described previously (33). For analysis of high molecular weight species, 4 μg of total RNA was loaded onto agarose denaturing gels (6% formaldehyde/1.2% agarose in HEPES-EDTA buffer) and electrophoresed for 4 h at 75 V. After washing, gels were transferred to nylon membranes by capillarity overnight in 10× saline sodium citrate (SSC). For analysis of low molecular weight species, 4 μg of total RNA was separated on denaturing acrylamide gels [8% acrylamide–bisacrylamide 19:1/8 M urea in Tris-borate-EDTA (TBE) buffer] for 2.5 h at 150 V, followed by semi-dry transfer in 0.5× TBE for 1 h at 150 mA. After UV cross-linking (120 mJ/cm^2^), membranes were pre-hybridized in 50% formamide, 5× SSPE, 5× Denhardt's solution, 1% w/v SDS, 200 μg/ml fish sperm DNA solution (Sigma) for 1 h at 65°C. The digoxigenin-labeled oligonucleotide probe was added and incubated for 1 h at 65°C and then overnight at 37°C. Sequences of northern blot probes are as follows: 5′-CTGCGAGGGAACCCCCAGCCGCGCA-3′ (ITS2); 5′-CAATGTGTCCTGCAATTCAC-3′ (5.8S-ITS2). After washing with 2× SSC, membranes were blocked in 1× blocking buffer (Roche) for 0.5 h at room temperature and incubated with anti-digoxigenin antibody (Roche, 1:10 000 dilution) for 0.5 h at room temperature, followed by washing steps (twice, each for 15 min) with washing buffer [0.1 M maleic acid, 0.15 M NaCl at pH 7.5, 0.3% Tween-20 (v/v)]. After equilibration in detection buffer (0.1 M Tris, 0.1 M NaCl at pH 9.5), membranes were incubated with chemiluminescent substrate CDP star® (Roche, 1:200 dilution) at room temperature for 10 min and then exposed to films.

### Translation assay

Global protein translation was measured by puromycin labeling as previously described (33). Briefly, cells were pre-treated with 10 μg/ml puromycin (Invitrogen) for 10 min. The cells were then harvested for IB detection of puromycylation of nascent peptides using anti-puromycin (clone 12D10; EMD Millipore).

### Cell proliferation assay

Cell viability was measured by 3-(4,5-dimethylthiazol-2-yl)-2,5-diphenyltetrazolium bromide (MTT) assays. Briefly, cells were incubated with 0.5 mg/ml MTT in medium for 3 h. After incubation, MTT medium was removed and dimethylsulfoxide (DMSO; 100 μl per well) was added to fully dissolve the purple formazan. The absorbance was measured at OD_560 nm_ and OD_690 nm_. The reduced absorbance (Abs_560 nm_ – Abs_6 90 nm_) represents the relative number of viable cells per well. For colony formation assays, an equal number of cells transfected with scrambled or EXOSC10 siRNA were cultured in DMEM containing 10% FBS for up to 2 weeks. The colonies were visualized by staining with 0.5% crystal violet in 50% ethanol.

## RESULTS

### USP36 associates with the nucleolar RNA exosome by directly binding to EXOSC10

**Figure 1.**
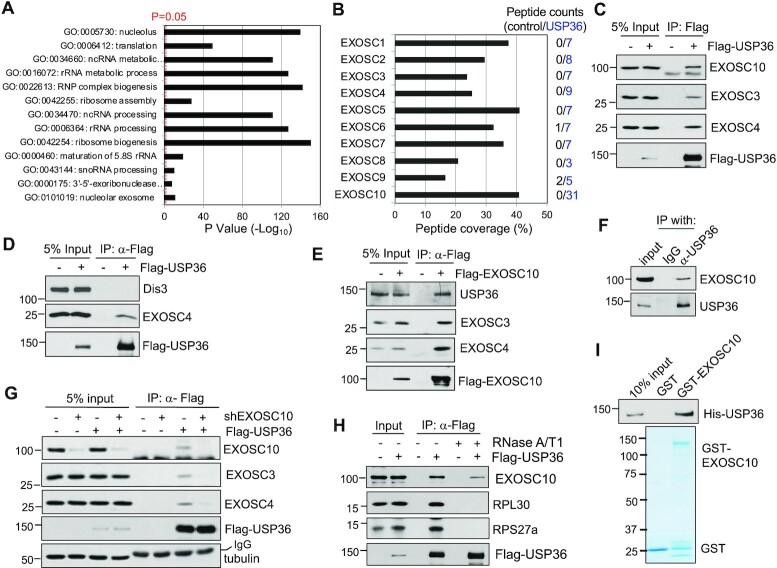
USP36 interacts with the RNA exosome. (**A**) GO analysis of the USP36-interacting proteins identified by MS analysis of anti-Flag immunoprecipitates from Flag-USP36-expressing 293 cells. A vertical dashed line represents the significance score, *P* = 0.05. (**B**) RNA exosome components revealed by MS analysis of immunoprecipitates from 293-Flag-USP36 cells by anti-Flag antibody. Shown are the percentiles of peptide coverage of each RNA exosome component with the numbers (blue) of peptides indicated on the right from Flag-USP36-expressing cells. The peptide numbers from control 293 cells are shown in black. (**C**) USP36 interacts with the RNA exosome. 293 cells transfected with Flag-USP36 were subjected to co-IP with anti-Flag antibody followed by IB. (**D**) USP36 does not interact with Dis3. H1299 cells were transfected with Flag-USP36 and assayed by co-IP using anti-Flag antibody followed by IB. (**E**) EXOSC10 interacts with endogenous USP36. Cell lysates from H1299 cells transfected with Flag-EXOSC10 were immunoprecipitated with anti-Flag antibody followed by IB. (**F**) Endogenous USP36 interacts with endogenous EXOSC10. 293 cell lysates were immunoprecipitated with anti-USP36 antibody (Proteintech) followed by IB with anti-EXOSC10. (**G**) Flag-USP36 associates with the RNA exosome through EXOSC10. H1299 cells were transfected with empty vector or Flag-USP36 and infected with scrambled or EXOSC10 shRNA lentiviruses, followed by co-IP with anti-Flag and IB. (**H**) Interaction between USP36 and EXOSC10 is partially dependent on RNA. 293 cells transfected with Flag-USP36 were subjected to co-IP with anti-Flag antibody in the presence or absence of 100 μg/ml RNase A and 100 U/ml RNase T1 treatment followed by IB. (**I**) USP36 directly interacts with EXOSC10 *in vitro*. Purified GST or GST–EXOSC10 immobilized on glutathione beads was incubated with purified His-USP36. Bound proteins were assayed by IB with anti-USP36 (top). Coomassie staining of GST and GST–EXOSC10 proteins is shown in the bottom panel.

To understand the mechanisms underlying the role of USP36 in ribosome biogenesis, we sought to identify USP36-interacting proteins. We performed an affinity purification of USP36-associated protein complexes from 293 cells stably expressing Flag-USP36 using anti-Flag antibody (M2) agarose gels, followed by elution with Flag peptides (Supplementary Figure S1C) and MS analysis. A total of 482 proteins were found in the eluates from Flag-USP36-expressing cells with higher abundance than in the control 293 cells (Supplementary Dataset S1). Gene Ontology (GO) analysis revealed that USP36-associated proteins are mainly enriched in the nucleolus and play critical roles in rRNA processing, snoRNA processing, ribosome assembly and translation (Figure 1A), highlighting the role of USP36 in ribosome biogenesis. Interestingly, all 10 components of the nucleolar RNA exosome, EXOSC1 to EXOSC10, but not Dis3, are present in the complexes (Figure 1B). Co-IP assays confirmed that Flag-USP36 binds to EXOSC10, the ‘cap’ subunit EXOSC3/hRrp40 and the ‘ring’ subunit EXOSC4/hRrp41 in 293 (Figure 1C) and HeLa (Supplementary Figure S1D) cells, but not with Dis3 (Figure 1D), the catalytical subunit of the RNA exosome in the nucleoplasm, consistent with the notion that USP36 interacts with the nucleolar RNA exosome. Flag-EXOSC10 also co-immunoprecipitated with USP36 using anti-Flag antibody (Figure 1E). Further, endogenous USP36 co-immunoprecipitated with endogenous EXOSC10 in both 293 (Figure 1F) and HeLa cells (Supplementary Figure S1E). To understand USP36 association with the RNA exosome in cells, we extracted the nuclear fraction of 293 cells transfected with Flag-USP36 and performed SEC. As shown in Supplementary Figure S1F, EXOSC10, EXOSC3 and EXOSC4 have the same elution peak, with the corresponding molecular weight of 400 kDa which is similar to the size of Exo10^hRrp6^. Interestingly, these RNA exosome proteins also co-elute with USP36, the exosome cofactor MTR4, ribosomal proteins L5, L11 and S27a, as well as ribosome biogenesis accessory factors such as NPM and Las1L in high molecular weight fractions (≥669 kDa), further supporting the association between USP36 and the exosome complex in pre-ribosome particles.

To further understand how USP36 interacts with the exosome, we tested whether this interaction is mediated by EXOSC10, as both peptide counts and peptide coverage of EXOSC10 in MS analysis are the highest among all the exosome subunits (Figure 1B) and it is among the top 20 binding proteins based on MS/MS spectral counts (Supplementary Table S1). As shown in Figure 1G, knockdown of EXOSC10 abrogated the interaction of USP36 with the exosome core components EXOSC3 and EXOSC4, suggesting that USP36 interaction with EXOSC10 is critical for its interaction with the core RNA exosome complex. Given that USP36 may interact with the exosome complex in pre-ribosome particles (Supplementary Figure S1F), we next examined whether the USP36–exosome interaction is dependent on RNA. We found that RNase treatment indeed reduced, but did not abolish, the interaction between USP36 and EXOSC10 in both 293 (Figure 1H) and HeLa cells (Supplementary Figure S1G), whereas the interaction of USP36 with RPL30 and RPS27a was abolished by the RNase treatment, suggesting that USP36 may directly interact with EXOSC10 and that this interaction is facilitated by RNA-containing pre-ribosome particles. We then tested whether USP36 directly interacts with EXOSC10 using GST PD assays. As shown in Figure 1I, recombinant His-USP36 protein purified from bacteria (Supplementary Figure S1H) was specifically bound by purified GST–EXOSC10, but not GST alone, indicating that USP36 directly interacts with EXOSC10 *in vitro*. Together, these results suggest that USP36 interacts with the RNA exosome via direct binding to EXOSC10 and this binding is facilitated by association with RNA.

### EXOSC10 and USP36 interact via their C-terminal domains

**Figure 2.**
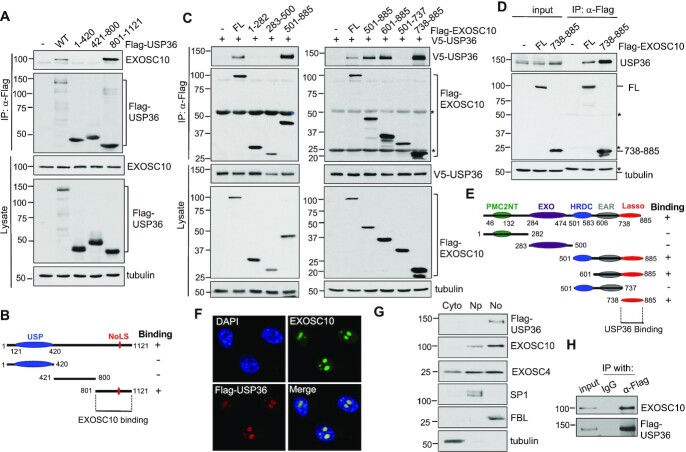
USP36 interacts with EXOSC10 via their C-terminal domains in the nucleolus. (**A** and **B**) EXOSC10 binds to the C-terminal domain of USP36. H1299 cells were transfected with Flag-USP36 or its deletion mutants as indicated, followed by co-IP with anti-Flag and IB analysis (A). The C-terminal EXOSC10-binding domain of USP36 is shown in (B). USP, ubiquitin-specific protease; NoLS, nucleolar localization signal. (**C–E**) USP36 binds to the Lasso region in the C-terminal domain (CTD) of EXOSC10. H1299 cells transfected with Flag-EXOSC10 or its deletion mutants together with (C) or without (D) V5-USP36 were subjected to co-IP with anti-Flag followed by IB. The diagram of EXOSC10 domains is shown in (E). PMC2NT, polycystin 2 N-terminal; EXO, exonuclease; HRDC, helicase and RNase D C-terminal; EAR, exosome-associated region. * indicates IgG. (**F**) Co-localization of Flag-USP36 and EXOSC10. HeLa cells transfected with Flag-USP36 were immunostained with anti-Flag (red) and anti-EXOSC10 (green). The nuclei were stained with DAPI (blue). (**G**) Subcellular distribution of Flag-USP36, EXOSC10 and EXOSC4 proteins. HeLa cells were fractionated into cytoplasmic (Cyto), nucleoplasmic (Np) and nucleolar fractions (No) followed by IB. Tubulin, SP1 and FBL were used as cytoplasmic, nucleoplasmic and nucleolar markers, respectively. (**H**) Co-IP of USP36 with EXOSC10 in the nucleolar fraction. Lysates from the nucleolar fraction shown in (G) were immunoprecipitated with anti-Flag antibody followed by IB with anti-EXOSC10 antibodies.

To understand how USP36 interacts with EXOSC10, we mapped the binding domains between USP36 and EXOSC10 using co-IP–IB assays. We first transfected cells with a panel of Flag-tagged USP36 deletion mutants followed by co-IP using anti-Flag antibody and IB with anti-EXOSC10 antibody. As shown in Figure 2A, the C-terminal nucleolar localization signal (NoLS)- (25,26) containing region (amino acids 801–1121), but not the N-terminal USP domain-containing (amino acids 1–420) and the middle (amino acids 421–800) regions, interacts with EXOSC10, indicating that EXOSC10 binds to the C-terminus of USP36 (Figure 2B). EXOSC10 contains multiple functional domains including the N-terminal PMC2NT, the central EXO and HRDC domains required for its exonuclease activity, as well as the C-terminal exosome-associated region (EAR) and recently characterized Lasso domains that bind to RNA substrates and stimulate the degradation and processing activities of exosome substrates (60,61). To examine which domain USP36 binds, we constructed a panel of Flag-tagged EXOSC10 deletion mutants. We co-introduced full-length EXOSC10 or its deletion mutants with V5-USP36 into H1299 cells and performed co-IP using anti-Flag antibody. As shown in Figure 2C, V5-USP36 specifically co-immunoprecipitated with the mutants containing the C-terminal Lasso domain (amino acids 738–885), but not the mutants lacking this region. Endogenous USP36 also co-immunoprecipitated with the C-terminal Lasso domain (Figure 2D). GST PD assays showed that the recombinant His-tagged Lasso domain of EXOSC10 protein purified from bacteria was specifically bound by the purified GST-fused C-terminus of USP36 (GST–USP36^801–1121^), but not by GST alone (Supplementary Figure S2A, B). Furthermore, RNase treatment also reduced, but did not abolish, the interaction of USP36 with the C-terminal Lasso domain of EXOSC10 (Supplementary Figure S2C) or the interaction of EXOSC10 with USP36^801–1121^ (Supplementary Figure S2D). Together, these results indicate that the C-terminal region of USP36 directly interacts with the C-terminal Lasso domain of EXOSC10 (Figure 2B, E) that can also be facilitated by the association with RNA.

### USP36 interacts with the RNA exosome in the nucleolus

USP36 is predominantly a nucleolar protein (26,29). Its C-terminal NoLS-containing region is required for interacting with EXOSC10 (Figure 2B). IF staining indicates that USP36 co-localizes with EXOSC10 in the nucleolus (Figure 2F). To further examine whether USP36 interacts with the RNA exosome in the nucleolus, we performed cell fractionation assays. As shown in Figure 2G, EXOSC10 is localized in both the nucleoplasm and the nucleolus, and the exosome core component EXOSC4 is located in both the cytoplasm and the nucleus, whereas USP36 is mainly localized in the nucleolus, consistent with the distribution of different RNA exosomes in different cell compartments (4). We performed co-IP assay using the lysates from the isolated nucleolar fraction and further confirmed that USP36 interacts with EXOSC10 in the nucleolus (Figure 2H). Thus, USP36 mainly associates with the nucleolar RNA exosome.

### USP36 SUMOylates EXOSC10 in cells and *in vitro*

**Figure 3.**
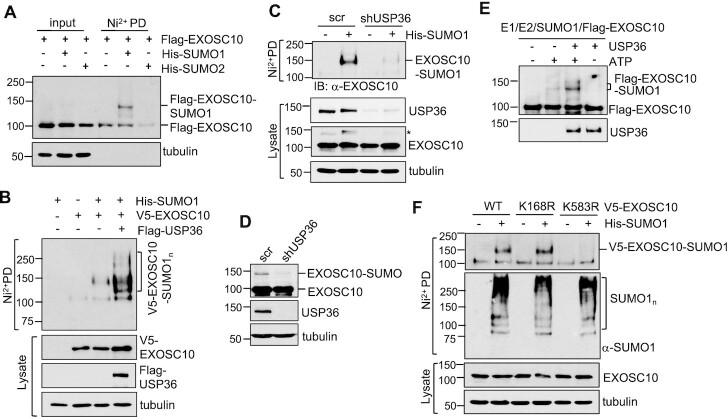
USP36 SUMOylates EXOSC10 in cells and *in vitro*. (**A**) EXOSC10 can be modified mainly by SUMO1. H1299 cells transfected with the indicated plasmids were subjected to Ni^2+^-NTA PD under denaturing conditions, followed by IB with anti-Flag antibody. (**B**) USP36 promotes SUMO1 modification of EXOSC10. H1299 cells transfected with the indicated plasmids were subjected to Ni^2+^-NTA PD followed by IB to detect EXOSC10 SUMOylation. The SUMO1-modified EXOSC10 is indicated. The protein expression is shown in the bottom panels. (**C**) Knockdown of USP36 attenuates SUMO1 modification of EXOSC10. HeLa cells transfected with His-SUMO1 and infected with scrambled (scr) or USP36 shRNA lentiviruses were subjected to Ni^2+^-NTA PD under denaturing conditions, followed by IB. * indicates SUMOylated EXOSC10 in cell lysates. (**D**) Knockdown of USP36 attenuates EXOSC10 modification by endogenous SUMO. HeLa cells infected with scr or USP36 shRNA lentiviruses were assayed by IB. (**E**) USP36 promotes SUMO1 modification of EXOSC10 *in vitro*. *In vitro* SUMOylation assays were performed with recombinant SUMO E1, Ubc9 (E2), SUMO1 and purified Flag-EXOSC10 with or without ATP and purified USP36 as indicated. (**F**) EXOSC10 is SUMOylated at K583. H1299 cells transfected with the indicated plasmids were subjected to Ni^2+^-NTA bead PD under denaturing conditions followed by IB.

We next asked whether USP36 regulates EXOSC10 ubiquitination as USP36 is a DUB (26,29). However, while we did observe the marginal ubiquitination of the exogenously expressed EXOSC10 that can be deubiquitinated by WT USP36, but not the catalytically inactive C131A mutant (Supplementary Figure S3A), the ubiquitination of endogenous EXOSC10 is undetectable (Supplementary Figure S3B), indicating that the steady-state levels of EXOSC10 ubiquitination under normal cell growth conditions is too low to be regulated by USP36. We recently found that USP36 possesses a novel SUMO ligase activity and mediates nucleolar protein group SUMOylation (33). Therefore, we examined whether USP36 promotes the SUMOylation of EXOSC10. H1299 cells were transfected with Flag-EXOSC10 together with control, His-SUMO1 or His-SUMO2 plasmid, followed by Ni^2+^-NTA PD assays under denaturing conditions and detection of SUMOylated EXOSC10 by IB. As shown in Figure 3A, EXOSC10 is mainly modified by SUMO1. Interestingly, USP36 markedly promoted EXOSC10 SUMOylation by both SUMO1 (Figure 3B) and SUMO2 (Supplementary Figure S3C). Consistently, knockdown of USP36 significantly reduced the levels of EXOSC10 SUMOylation in cells by both exogenously expressed SUMO1 as determined by Ni^2+^-NTA PD assays (Figure 3C) and endogenous SUMO shown as the modified EXOSC10 band in IB assays (Figure 3D). This modified band is the SUMO-modified EXOSC10 as it was also abolished by knockdown of either SUMO E1 subunit SAE2 or Ubc9 (Supplementary Figure S3D) and can be immunoprecipitated by using anti-EXOSC10 antibody and detected by anti-SUMO1 antibody (Supplementary Figure S3E). To test whether USP36 directly SUMOylates EXOSC10, we performed *in vitro* SUMOylation assays using recombinant proteins. As shown in Figure 3E, *in vitro* SUMOylation reaction using recombinant E1 (SAE1/SAE2), E2 (Ubc9), SUMO1 and ATP resulted in marginal SUMO conjugation of EXOSC10, consistent with the notion that SUMO E1 and Ubc9 can directly mediate SUMOylation *in vitro*, although less efficiently (62,63). Notably, USP36 markedly increased EXOSC10 SUMOylation by SUMO1 *in vitro*, demonstrating that USP36 acts as a SUMO E3 for EXOSC10. To map the SUMOylation sites in EXOSC10, we mutated the consensus SUMO lysines, K168 and K583, predicted by the GPS-SUMO tool (64), as well as other reported SUMO lysines by high-throughput proteomic analysis including K19, K710, K826, K833, K859 and K873 (49,53,65,66), to Arg (R) and examined their SUMOylation in cells. As shown in Figure 3F and Supplementary Figure S3F and G, mutating K583, but not K168, K19, K710, K826, K833, K859 or K873, abolished EXOSC10 SUMOylation, indicating that K583 is the predominant acceptor Lys for EXOSC10 SUMOylation. Of note, mutating K583 did not affect the ubiquitination of EXOSC10 (Supplementary Figure S3H), suggesting that K583 is not subjected to the ubiquitination modification.

### USP36 does not significantly affect the levels of EXOSC10 and its association with the RNA exosome

To understand whether USP36-mediated SUMOylation affects EXOSC10 protein levels, we performed USP36 knockdown experiments. As shown in Supplementary Figure S4A, knockdown of USP36 did not reduce the levels of EXOSC10 or other tested exosome core subunits including EXOSC2, EXOSC3 and EXOSC4. These exosome proteins are highly stable (Supplementary Figure S4B, C). Knockdown of USP36 did not reduce the half-life of EXOSC10 (Supplementary Figure S4D). These results suggest that EXOSC10 is not mainly regulated by ubiquitination-mediated proteasome degradation and that USP36-mediated SUMOylation of EXOSC10 does not significantly regulate its protein levels.

To examine whether USP36-mediated SUMOylation regulates EXOSC10 localization and association with the RNA exosome in the nucleolus, we performed IF staining in cells with or without USP36 knockdown. As shown in Supplementary Figure S4E, knockdown of USP36 did not apparently alter the predominant nucleolar localization of EXOSC10 in cells. Also, mutating the K583 SUMO site did not alter the nucleolar localization of EXOSC10 in HeLa (Supplementary Figure S5A) and U2OS (Supplementary Figure S5B) cells. Knockdown of USP36 also does not interfere with the association of EXOSC10 with the core exosome subunit EXOSC4 as determined by co-IP assays using anti-EXOSC10 antibody (Supplementary Figure S5C). Furthermore, the K583R mutant binds to the exosome as efficiently as WT EXOSC10 as probed by the presence of the core exosome subunits EXOSC3 and EXOSC4 (Supplementary Figure S5D). Also, the K583R mutation does not affect the interaction of EXOSC10 with USP36 (Supplementary Figure S5E). Thus, USP36-mediated SUMOylation does not significantly affect the nucleolar localization of EXOSC10 and its association with core exosome and USP36.

### Ablation of EXOSC10 SUMOylation inhibits rRNA processing

**Figure 4.**
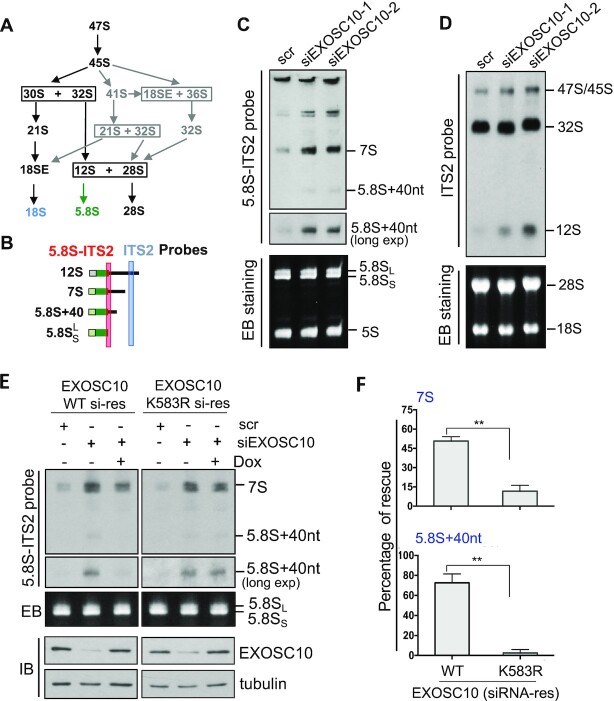
SUMOylation of EXOSC10 plays a critical role in pre-rRNA processing. (**A**) Diagram showing the intermediate rRNA products of the major (black) and minor (gray) pre-rRNA processing pathways. (**B**) Diagram of the 12S rRNA processing. The positions of ITS2 and 5.8S–ITS2 junction probes for northern blot are indicated. (**C** and **D**) EXOSC10 is required for 12S rRNA processing. HeLa cells transfected with scr or two different EXOSC10 siRNAs were assayed for rRNA processing by northern blot (top panels) using the 5.8S–ITS2 junction (C) and ITS2 (D) probes as indicated. Ethidium bromide (EB) staining of the RNA gels is shown in the bottom panels. (**E** and **F**) WT EXOSC10, but not the K583R mutant, rescued EXOSC10 depletion-induced attenuation of 12S rRNA processing. HeLa cells stably expressing Dox-inducible siRNA-resistant EXOSC10 (WT or the K583R mutant) were transfected with scr or EXOSC10 siRNAs and treated with or without Dox. Total RNAs extracted from the cells were assayed by northern blot using the 5.8S–ITS2 junction probe and IB detection of the expression of EXOSC10 (E). The 7S and 5.8S + 40 nt species were quantified and the rescue efficiency is calculated from three independent experiments (F). **P<0.01, comparison between WT and the K583 mutant as determined by Student's *t*-test.

Next, we sought to examine whether USP36-mediated SUMOylation of EXOSC10 regulates the nucleolar RNA exosome function in rRNA processing. The 47S pre-rRNA is processed to mature 18S, 5.8S and 28S rRNAs via multiple steps of processing cleavage, as illustrated in Figure 4A. The RNA exosome has been shown to play a key role in the processing of the 3′ end of 5.8S (from 12S intermediates to 5.8S, Figure 4B) and 18S (from 21S to 18SE) precursors (7,16,67) as well as the degradation of the 5′-ETS (15). Using northern blot with a probe hybridizing to the 5.8S–ITS2 conjunction (Figure 4C) and the ITS2 (Figure 4D), respectively, we confirmed that knockdown of EXOSC10 by two different siRNAs markedly impaired rRNA processing, leading to accumulation of 5.8S precursors including 12S, 7S and 5.8S + 40 nt (Figure 4C, D). To examine the role of EXOSC10 SUMOylation in rRNA processing, we performed EXOSC10 knockdown and rescue experiments. We established HeLa cells stably expressing Tet-inducible siRNA-resistant EXOSC10 (WT or the K583R mutant), whose expression can be induced by Dox. These cells were transfected with control scr RNA or EXOSC10 siRNA in the absence or presence of Dox and then assayed by northern blot using a 5.8S–ITS2 probe to monitor the 12S rRNA processing. As shown in Figure 4E and summarized in Figure 4F, the accumulation of 7S and 5.8S + 40 nt rRNA upon EXOSC10 knockdown was markedly alleviated by the Dox-induced expression of siRNA-resistant WT EXOSC10, but not the K583R mutant. Together, these data suggest that EXOSC10 SUMOylation by USP36 plays a critical role in rRNA processing. USP36 is critical for multiple steps of rRNA processing and its depletion results in the reduction of both 21S and 12S rRNA species (27, 33). Consistently, we showed that knockdown of USP36 significantly reduced the levels of 12S rRNA (Supplementary Figure S6A, B). Further analysis showed that knockdown of USP36 markedly impaired 12S rRNA processing, as indicated by the accumulation of 7S species and the marked increase of the ratios of 7S and 5.8S + 40 nt species to 12S rRNA precursors (Supplementary Figure S6C, D). These results suggest that USP36 plays an important role in 5.8S rRNA maturation, correlating with its role in regulating EXOSC10 and RNA exosome function in processing 5.8S precursors.

### Ablation of EXOSC10 SUMOylation inhibits its binding to pre-rRNA

**Figure 5.**
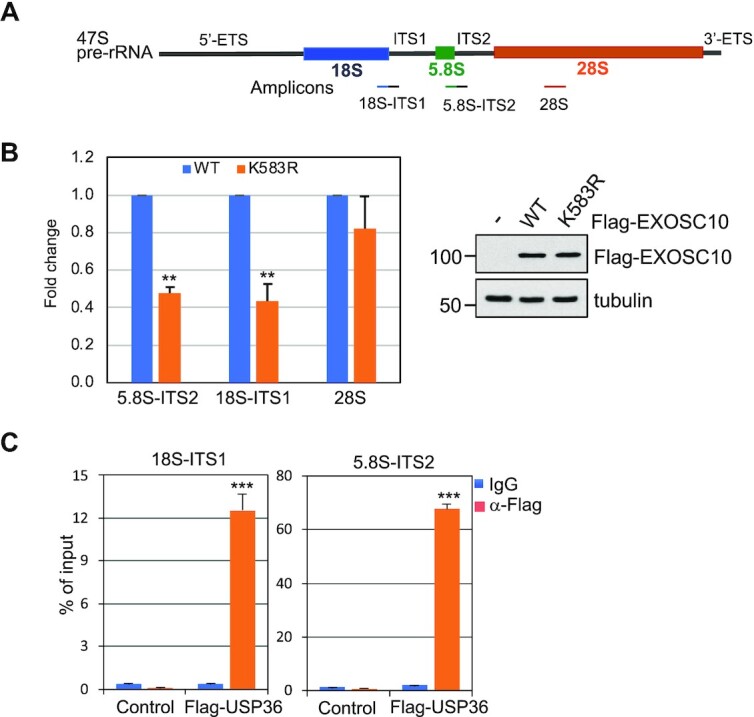
The SUMOylation of EXOSC10 is critical for its binding to pre-rRNA. (**A**) Diagram of 47S pre-rRNA showing the primers used for RT–qPCR assays indicated in the bottom. (**B**) Mutating K583 impairs the binding of EXOSC10 to rRNA precursors. 293 cells transfected with control or Flag-EXOSC10 (WT and the K583R mutant) plasmids were subjected to RNA-IP with anti-Flag followed by RT–qPCR. Shown is the fold reduction of relative rRNA binding of EXOSC10^K583R^ compared with EXOSC10^WT^ and normalized to empty vector-transfected cells from five independent experiments. The relative RNA enrichment was calculated by dividing RNAs in anti-Flag immunoprecipitates by that in control IgG immunoprecipitates. **P<0.01, compared with Flag-EXOSC10^WT^-transfected cells. *P*-values were calculated by Student's *t*-test. The expression of Flag-EXOSC10^WT^ and Flag-EXOSC10^K583R^ assayed by IB is shown on the right. (**C**) USP36 binds to pre-rRNA. HeLa cells transfected with control or Flag-USP36 were subjected to RNA-IP using anti-Flag or control mouse IgG, followed by RT–qPCR analysis. Shown are percentage enrichment relative to input from three independent experiments. *P*-values were calculated by Student's *t*-test. ***P<0.001.

To understand how USP36-mediated SUMOylation may regulate EXOSC10’s activity in rRNA processing, we tested whether USP36-mediated SUMOylation promotes EXOSC10 binding to rRNA. To this end, we performed RNA-IP assays followed by RT–qPCR assays using primers amplifying different regions of pre-rRNA (Figure 5A). As shown in Figure 5B, mutating K583 significantly attenuated the binding of EXOSC10 to pre-rRNA across the 5.8S–ITS2 junction and 18S–ITS1 junction, consistent with the role of EXOSC10 in processing the 5.8S and 18S precursors. These data suggest that USP36-mediated EXOSC10 SUMOylation promotes the targeting of EXOSC10 to pre-rRNAs. Of note, although EXOSC10 binds to 28S as reported by CLIP-seq assays (17), mutating K583 only slightly, but not significantly, reduced the binding of EXOSC10 to mature 28S rRNA (Figure 5B), suggesting that EXOSC10 SUMOylation may specifically affect the exosome-mediated maturation of 5.8S and 18S rRNA. We also examined whether USP36 could regulate the exosome function by binding to rRNA precursors. Indeed, RNA-IP assays showed that USP36 strongly binds to pre-rRNAs (Figure 5C), suggesting that USP36 itself may act as a pre-rRNA-binding protein or associate with pre-rRNA by binding to the exosome in pre-ribosome particles to regulate exosome function at the pre-rRNA.

### Ablation of EXOSC10 SUMOylation inhibits translation and suppresses cell proliferation

**Figure 6.**
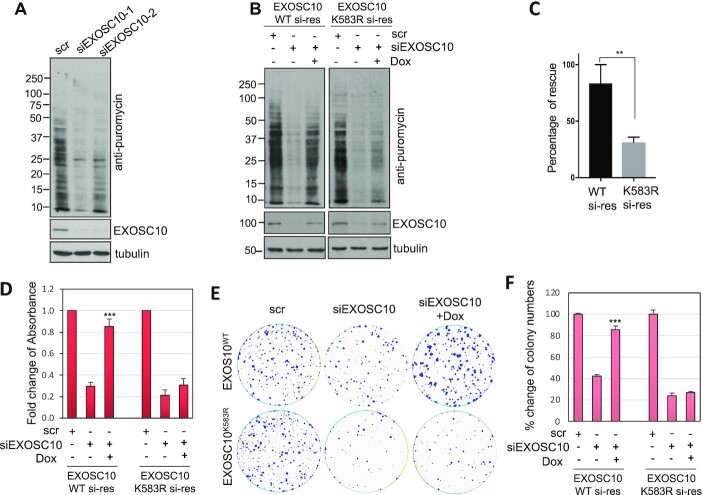
SUMOylation of EXOSC10 plays a critical role in protein translation and cell growth. (**A**) Knockdown of EXOSC10 inhibits global protein translation. HeLa cells transfected with scr or one of the two EXOSC10 siRNAs were pulse-labeled with puromycin followed by IB with anti-puromycin to detect new protein synthesis. (**B** and **C**) The K583R mutant of EXOSC10 rescues the translation inhibition by endogenous EXOSC10 depletion less efficiently compared with WT EXOSC10. HeLa cells stably expressing Dox-inducible siRNA-resistant WT EXOSC10 or the K583R mutant were transfected with scr or EXOSC10 siRNAs and treated with or without Dox, followed by puromycin labeling and IB analysis (B). The percentage rescue is quantified from three independent experiments (C). Data were presented as mean ± standard deviation (SD). The *P*-value was determined by Student's *t*-test. **P<0.01. (**D**–**F**) The K583R mutant rescues the growth inhibition by knockdown of endogenous EXOSC10 less efficiently compared with WT EXOSC10. HeLa cells stably expressing Dox-inducible siRNA-resistant WT EXOSC10 or the K583R mutant were transfected with scr or EXOSC10 siRNAs and treated with or without Dox, followed by MTT cell proliferation (D) and colony formation (E, F) assays. Shown are the fold changes of absorbance from four independent experiments (D), one representative colony formation (E) and the average percentage changes of the colony numbers (F) from three independent experiments. *P*-values shown were calculated by Student's *t*-test. ***P<0.001, compared with cells transfected with EXOSC10 siRNAs without induction of siRNA-resistant EXOSC10.

Given the critical role of EXOSC10 SUMOylation in rRNA processing, we next sought to examine the role of USP36-mediated EXOSC10 SUMOylation in protein translation and cell growth. To do so, we first measured nascent peptide synthesis using puromycin pulse labeling in cells followed by IB with anti-puromycin antibody. As shown in Figure 6A, knockdown of EXOSC10 by two different siRNAs markedly inhibited protein translation. Consistent with the role of EXOSC10 SUMOylation in rRNA processing (Figure 4), Dox-induced expression of WT EXOSC10, but not the SUMOylation-defective K583R mutant, largely restored the protein translation (Figure 6B, C). Cell proliferation assays measured by MTT (Figure 6D) and colony formation (Figure 6E, F) assays also showed that Dox-induced expression of WT EXOSC10, but not the K583R mutant, markedly rescued cell growth inhibition by knockdown of endogenous EXOSC10, demonstrating that EXOSC10 SUMOylation at K583 plays a critical role in protein translation and cell proliferation.

### Ribosomal stress attenuates EXOSC10 SUMOylation by reducing USP36

**Figure 7.**
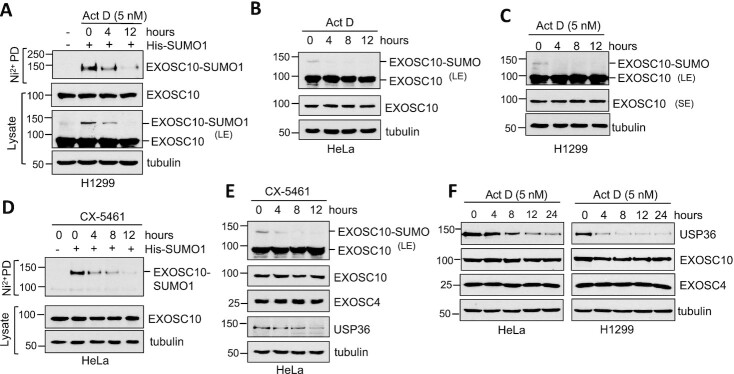
EXOSC10 SUMOylation and USP36 expression are reduced in cells following the perturbation of ribosome biogenesis. (**A**) EXOSC10 SUMOylation is reduced in cells in response to the treatment with a low dose of Act D. H1299 cells transfected with or without His-SUMO1 were treated with or without 5 nM Act D for different times, followed by Ni^2+^-NTA PD under denaturing conditions to detect SUMOylated EXOSC10 by IB. The protein expression is shown in the bottom panels. (**B** and **C**) EXOSC10 modified by endogenous SUMO is also reduced in cells in response to the low dose Act D treatment. HeLa (B) and H1299 (C) cells were treated with or without 5 nM Act D for different times, followed by IB. SUMOylated EXOSC10 is shown in IB with longer exposure (LE) in the top panels. (**D**) EXOSC10 SUMOylation is reduced in cells by the treatment with CX-5461. HeLa cells transfected with or without His-SUMO1 were treated with or without 1 μM CX-5461 for different times, followed by Ni^2+^-NTA PD under denaturing conditions to detect SUMOylated EXOSC10 by IB. The protein expression is shown in the bottom panels. (**E**) CX-5461 treatment reduces USP36 levels and attenuates EXOSC10 modification by endogenous SUMO. HeLa cells treated with 1 μM CX-5461 for different times were assayed by IB. (**F**) The level of USP36, but not of EXOSC10 and the core exosome component EXOSC4, is reduced by the low dose Act D treatment. HeLa and H1299 cells treated with 5 nM Act D for different times were analyzed by IB.

To further understand the role of USP36-mediated EXOSC10 SUMOylation in ribosome biogenesis, we asked whether EXOSC10 SUMOylation could be regulated in cells in response to ribosomal stress caused by the perturbation of ribosome biogenesis. We first treated cells with a low dose (5 nM) of Act D, which specifically inhibits rRNA synthesis and causes ribosome stress (56,59,68), and examined the EXOSC10 SUMOylation. Ni^2+^-NTA PD assays showed that Act D treatment markedly reduced the levels of EXOSC10 SUMOylation by exogenously expressed SUMO-1, but not the total levels of EXOSC10, in a time-dependent manner (Figure 7A). EXOSC10 SUMOylation by endogenous SUMO shown as the modified EXOSC10 band in IB is also significantly inhibited by the treatment with Act D in both HeLa and H1299 cells (Figure 7B, C). To further validate the reduction of EXOSC10 SUMOylation in response to ribosomal stress, we treated cells with the small molecule RNA Pol I inhibitor CX-5461 (69). Indeed, CX-5461 treatment also significantly impaired EXOSC10 SUMOylation by both exogenously expressed SUMO1 (Figure 7D) and endogenous SUMO (Figure 7E) in HeLa cells. Similar results were also observed in other tested cell lines including H1299 cells (Supplementary Figure S7A, B). Interestingly, both Act D treatment and CX-5461 treatment significantly reduced the levels of USP36 in HeLa, H1299 (Figure 7E, F; Supplementary Figure S7B) and U2OS cells (Supplementary Figure S7C), whereas the levels of EXOSC10 and the examined core exosome subunit EXOSC4 were not changed by the Act D or CX-5461 treatment (Figure 7E, F; Supplementary Figure S7B), indicating that the attenuated EXOSC10 SUMOylation following ribosomal stress is associated with the reduction of USP36 protein. Together, these results suggest that the levels of USP36 and EXOSC10 SUMOylation are reduced in cells in response to ribosomal stress, thus coordinating with ribosome biogenesis.

## DISCUSSION

The RNA exosome plays a critical role in RNA processing, degradation and quality control, thus being essential for normal cell growth and proliferation. The nucleolar RNA exosome is critical for ribosome biogenesis by processing pre-rRNAs and snoRNAs (16,17). Although Rrp6 is the only non-essential component among the 11 exosome subunits for yeast cell growth, human EXOSC10 (hRrp6) is essential for cell growth (17). EXOSC10 knockout mice are embryonic lethal, with embryogenesis arrested at the morula stage (70), suggesting that EXOSC10 plays an indispensable role in early embryogenesis and animal development. As ribosome biogenesis is a complex and dynamic cellular process and is subjected to extensive regulation in response to growth signals and cellular stressors, it is conceivable that the function of the nucleolar RNA exosome is also highly regulated.

In this study, we report that the key RNA exosome component EXOSC10 is SUMOylated at K583 by USP36 in the nucleolus. USP36-mediated EXOSC10 SUMOylation is critical for the nucleolar RNA exosome function in rRNA processing and cell growth, as the SUMO-defective K583R mutant of EXOSC10 has significantly impaired function to rescue the growth inhibition and pre-rRNA processing impairment induced by knockdown of endogenous EXOSC10 (Figures 4 and 6), and knockdown of USP36 results in similar defects in the processing of 5.8S rRNA precursors to that by the knockdown of EXOSC10 (Supplementary Figure S6). We further show that the K583R mutant of EXOSC10 has impaired pre-rRNA binding activity (Figure 5). As USP36 does not significantly alter the protein levels and cellular localization of the exosome proteins, the reduced binding of the K583R mutant to pre-rRNA might be due to the impairment of conformational change of EXOSC10 upon SUMOylation. The K583 residue is located close to the C-terminal Lasso motif (61), which is critical for binding to RNAs. Thus, K583 SUMOylation could facilitate a conformational change of EXOSC10 that favors its high-affinity binding to pre-rRNA. It has been suggested that the exosome can be recruited to different pre-ribosome particles via the binding of the TRAMP complex component MTR4 to the adaptor proteins such as Nop53 and UTP18 (5,15). Both Nop53 and UTP18 as well as MTR4 were present in our purified Flag-USP36-associated protein complexes as determined by MS analysis (Supplementary Dataset 1; Supplementary Table S1). We indeed observed that USP36 interacts with the TRAMP complex (data not shown). Therefore, it is also possible that EXOSC10 SUMOylation by USP36 could facilitate the assembly and stabilization of the exosome–TRAMP–adaptor protein complexes in the small subunit processome (SSU) and the large subunit processome (LSU). Accumulating evidence suggests that SUMOylation tends to target a group of functionally and physically connected proteins, called protein group SUMOylation (35,71), which allows multiple SUMO–SIM (SUMO-interacting motif) interactions that contribute to the formation and stabilization of the multi-protein complexes (35,71). Previous proteomics analyses observed the SUMOylation of the TRAMP complex proteins ZCCHC7 and PAPD5 (49,66,72–76). The TRAMP complex proteins each contain multiple putative SIMs predicted by the GPS-SUMO tool (64) (not shown). Thus, it is interesting to examine whether USP36 also SUMOylates the Nop53, UTP18 and TRAMP components, given that they all physically interact with USP36, thus mediating exosome–TRAMP–adaptor protein group SUMOylation to promote and/or stabilize the exosome–TRAMP–adaptor protein complex in pre-ribosome particles via multiple SUMO–SIM interactions. Together, our data reveal that USP36 is a novel regulator of the nucleolar RNA exosome by acting as a SUMO ligase to mediate EXOSC10 SUMOylation. Knight *et al.* (77) previously reported that EXOSC10 SUMOylation is increased along with global SUMOylation in cells in response to cooling, resulting in reduced levels of EXOSC10 and defects in ribosome biogenesis. However, it is not clear whether such SUMOylation is the consequence or the cause of changes in cells under cooling conditions and whether abolishing EXOSC10 SUMOylation could alleviate cooling-mediated suppression of rRNA processing and ribosome biogenesis. Compared with mutating three lysine residues, K168, K201 and K583, used in the study (77), we showed that mutating K583 alone can abolish EXOSC10 SUMOylation without affecting EXOSC10 levels. Furthermore, we have shown that EXOSC10 SUMOylation is markedly reduced in cells in response to the perturbation of ribosome biogenesis mediated by treatment with a low dose of Act D or the RNA Pol I-specific inhibitor CX-5461 (Figure 7), suggesting that EXOSC10 SUMOylation is tightly regulated and coordinated with ribosome biogenesis.

EXOSC10 and the RNA exosome core subunit proteins are all stable proteins and their levels are not significantly affected by either overexpression or knockdown of USP36. This is similar to the regulation of the snoRNP complex by USP36 that mainly functions to mediate snoRNP SUMOylation, but not ubiquitination (33). Consistently, the steady-state levels of EXOSC10 ubiquitination are below the level of detection (Supplementary Figure S3B), suggesting that under normal conditions, USP36 mainly acts to SUMOylate EXOSC10 to regulate its function, but not its levels, whereas its DUB activity may play a role in maintaining RNA exosome subunit proteins assembled in the RNA exosome in their deubiquitinated state in the nucleolus.

Interestingly, we also found that USP36 associates with pre-rRNA (Figure 5C) and snoRNAs (not shown), indicating that USP36 itself could be an RNA-binding protein. A previous study using microRNA (miRNA) screening of RNA-binding protein has shown that USP36 is able to bind to several miRNA precursors (78). Thus, our finding reveals an additional mechanism underlying USP36’s role in ribosome biogenesis: binding to pre-rRNA and mediating rRNA processing, including its critical role in regulating the RNA exosome function to process 5.8S and 18S rRNA. Future studies would aim to evaluate whether USP36 directly binds to pre-rRNA or indirectly through its association with the exosome and other interacting proteins in pre-ribosome particles. As our proteomic data showed that USP36 forms large protein complexes by associating with many ribosome biogenesis-related proteins and that the complex may contain additional RNAs, such as pre-rRNA and snoRNAs, USP36 may be critical for the nucleolar protein–RNA complex formation via its DUB-, SUMO E3- and RNA-binding activities. Thus, USP36 is a multi-functional nucleolar protein and may play a central role in ribosome biogenesis and translation by acting as a central regulatory hub for nucleolar protein dynamics and ribosome biogenesis.

## DATA AVAILABILITY

The mass spectrometry proteomics data have been deposited in the ProteomeXchange Consortium via the PRIDE partner repository with the dataset identifier PXD031592.

## Supplementary Material

gkad140_Supplemental_Files

## References

[1] Kressler D. , Hurt E., Bassler J. A puzzle of life: crafting ribosomal subunits. Trends Biochem. Sci. 2017; 42:640–654. 28579196 10.1016/j.tibs.2017.05.005

[2] Rodnina M.V. , Wintermeyer W. Recent mechanistic insights into eukaryotic ribosomes. Curr. Opin. Cell Biol. 2009; 21:435–443. 19243929 10.1016/j.ceb.2009.01.023

[3] Januszyk K. , Lima C.D. The eukaryotic RNA exosome. Curr. Opin. Struct. Biol. 2014; 24:132–140. 24525139 10.1016/j.sbi.2014.01.011 PMC3985421

[4] Kilchert C. , Wittmann S., Vasiljeva L. The regulation and functions of the nuclear RNA exosome complex. Nat. Rev. Mol. Cell Biol. 2016; 17:227–239. 26726035 10.1038/nrm.2015.15

[5] Zinder J.C. , Lima C.D. Targeting RNA for processing or destruction by the eukaryotic RNA exosome and its cofactors. Genes Dev. 2017; 31:88–100. 28202538 10.1101/gad.294769.116 PMC5322736

[6] Allmang C. , Kufel J., Chanfreau G., Mitchell P., Petfalski E., Tollervey D Functions of the exosome in rRNA, snoRNA and snRNA synthesis. EMBO J. 1999; 18:5399–5410. 10508172 10.1093/emboj/18.19.5399 PMC1171609

[7] Briggs M.W. , Burkard K.T., Butler J.S. Rrp6p, the yeast homologue of the human PM-Scl 100-kDa autoantigen, is essential for efficient 5.8 S rRNA 3' end formation. J. Biol. Chem. 1998; 273:13255–13263. 9582370 10.1074/jbc.273.21.13255

[8] Mitchell P. , Petfalski E., Shevchenko A., Mann M., Tollervey D The exosome: a conserved eukaryotic RNA processing complex containing multiple 3'→5' exoribonucleases. Cell. 1997; 91:457–466. 9390555 10.1016/s0092-8674(00)80432-8

[9] Schneider C. , Kudla G., Wlotzka W., Tuck A., Tollervey D Transcriptome-wide analysis of exosome targets. Mol. Cell. 2012; 48:422–433. 23000172 10.1016/j.molcel.2012.08.013 PMC3526797

[10] Makino D.L. , Baumgartner M., Conti E. Crystal structure of an RNA-bound 11-subunit eukaryotic exosome complex. Nature. 2013; 495:70–75. 23376952 10.1038/nature11870

[11] Makino D.L. , Schuch B., Stegmann E., Baumgartner M., Basquin C., Conti E. RNA degradation paths in a 12-subunit nuclear exosome complex. Nature. 2015; 524:54–58. 26222026 10.1038/nature14865

[12] Wasmuth E.V. , Januszyk K., Lima C.D. Structure of an Rrp6–RNA exosome complex bound to poly(A) RNA. Nature. 2014; 511:435–439. 25043052 10.1038/nature13406 PMC4310248

[13] Staals R.H. , Bronkhorst A.W., Schilders G., Slomovic S., Schuster G., Heck A.J., Raijmakers R., Pruijn G.J. Dis3-like 1: a novel exoribonuclease associated with the human exosome. EMBO J. 2010; 29:2358–2367. 20531389 10.1038/emboj.2010.122 PMC2910272

[14] Tomecki R. , Kristiansen M.S., Lykke-Andersen S., Chlebowski A., Larsen K.M., Szczesny R.J., Drazkowska K., Pastula A., Andersen J.S., Stepien P.P. et al. The human core exosome interacts with differentially localized processive RNases: hDIS3 and hDIS3L. EMBO J. 2010; 29:2342–2357. 20531386 10.1038/emboj.2010.121 PMC2910271

[15] Thoms M. , Thomson E., Bassler J., Gnadig M., Griesel S., Hurt E. The exosome is recruited to RNA substrates through specific adaptor proteins. Cell. 2015; 162:1029–1038. 26317469 10.1016/j.cell.2015.07.060

[16] Tafforeau L. , Zorbas C., Langhendries J.L., Mullineux S.T., Stamatopoulou V., Mullier R., Wacheul L., Lafontaine D.L. The complexity of human ribosome biogenesis revealed by systematic nucleolar screening of pre-rRNA processing factors. Mol. Cell. 2013; 51:539–551. 23973377 10.1016/j.molcel.2013.08.011

[17] Davidson L. , Francis L., Cordiner R.A., Eaton J.D., Estell C., Macias S., Caceres J.F., West S. Rapid depletion of DIS3, EXOSC10, or XRN2 reveals the immediate impact of exoribonucleolysis on nuclear RNA metabolism and transcriptional control. Cell Rep. 2019; 26:2779–2791. 30840897 10.1016/j.celrep.2019.02.012 PMC6403362

[18] Halbach F. , Reichelt P., Rode M., Conti E. The yeast ski complex: crystal structure and RNA channeling to the exosome complex. Cell. 2013; 154:814–826. 23953113 10.1016/j.cell.2013.07.017

[19] Hardwick S.W. , Luisi B.F. Rarely at rest: RNA helicases and their busy contributions to RNA degradation, regulation and quality control. RNA Biol. 2013; 10:56–70. 23064154 10.4161/rna.22270 PMC3590238

[20] Jia H. , Wang X., Liu F., Guenther U.P., Srinivasan S., Anderson J.T., Jankowsky E. The RNA helicase Mtr4p modulates polyadenylation in the TRAMP complex. Cell. 2011; 145:890–901. 21663793 10.1016/j.cell.2011.05.010 PMC3115544

[21] LaCava J. , Houseley J., Saveanu C., Petfalski E., Thompson E., Jacquier A., Tollervey D RNA degradation by the exosome is promoted by a nuclear polyadenylation complex. Cell. 2005; 121:713–724. 15935758 10.1016/j.cell.2005.04.029

[22] Vanacova S. , Wolf J., Martin G., Blank D., Dettwiler S., Friedlein A., Langen H., Keith G., Keller W. A new yeast poly(A) polymerase complex involved in RNA quality control. PLoS Biol. 2005; 3:e189. 15828860 10.1371/journal.pbio.0030189 PMC1079787

[23] Wyers F. , Rougemaille M., Badis G., Rousselle J.C., Dufour M.E., Boulay J., Regnault B., Devaux F., Namane A., Seraphin B. et al. Cryptic pol II transcripts are degraded by a nuclear quality control pathway involving a new poly(A) polymerase. Cell. 2005; 121:725–737. 15935759 10.1016/j.cell.2005.04.030

[24] Lubas M. , Christensen M.S., Kristiansen M.S., Domanski M., Falkenby L.G., Lykke-Andersen S., Andersen J.S., Dziembowski A., Jensen T.H. Interaction profiling identifies the human nuclear exosome targeting complex. Mol. Cell. 2011; 43:624–637. 21855801 10.1016/j.molcel.2011.06.028

[25] Endo A. , Kitamura N., Komada M. Nucleophosmin/B23 regulates ubiquitin dynamics in nucleoli by recruiting deubiquitylating enzyme USP36. J. Biol. Chem. 2009; 284:27918–27923. 19679658 10.1074/jbc.M109.037218 PMC2788843

[26] Endo A. , Matsumoto M., Inada T., Yamamoto A., Nakayama K.I., Kitamura N., Komada M. Nucleolar structure and function are regulated by the deubiquitylating enzyme USP36. J. Cell Sci. 2009; 122:678–686. 19208757 10.1242/jcs.044461

[27] Fraile J.M. , Campos-Iglesias D., Rodriguez F., Astudillo A., Vilarrasa-Blasi R., Verdaguer-Dot N., Prado M.A., Paulo J.A., Gygi S.P., Martin-Subero J.I. et al. Loss of the deubiquitinase USP36 destabilizes the RNA helicase DHX33 and causes preimplantation lethality in mice. J. Biol. Chem. 2018; 293:2183–2194. 29273634 10.1074/jbc.M117.788430 PMC5808777

[28] Richardson L.A. , Reed B.J., Charette J.M., Freed E.F., Fredrickson E.K., Locke M.N., Baserga S.J., Gardner R.G. A conserved deubiquitinating enzyme controls cell growth by regulating RNA polymerase I stability. Cell Rep. 2012; 2:372–385. 22902402 10.1016/j.celrep.2012.07.009 PMC3638920

[29] Sun X.X. , He X., Yin L., Komada M., Sears R.C., Dai M.S. The nucleolar ubiquitin-specific protease USP36 deubiquitinates and stabilizes c-Myc. Proc. Natl Acad. Sci. USA. 2015; 112:3734–3739. 25775507 10.1073/pnas.1411713112 PMC4378440

[30] van Riggelen J. , Yetil A., Felsher D.W. MYC as a regulator of ribosome biogenesis and protein synthesis. Nat. Rev. Cancer. 2010; 10:301–309. 20332779 10.1038/nrc2819

[31] White R.J. RNA polymerases I and III, growth control and cancer. Nat. Rev. Mol. Cell Biol. 2005; 6:69–78. 15688068 10.1038/nrm1551

[32] Sun X.X. , Sears R.C., Dai M.S. Deubiquitinating c-Myc: USP36 steps up in the nucleolus. Cell Cycle. 2015; 14:3786–3793. 26697836 10.1080/15384101.2015.1093713 PMC4825782

[33] Ryu H. , Sun X.X., Chen Y., Li Y., Wang X., Dai R.S., Zhu H.M., Klimek J., David L., Fedorov L.M. et al. The deubiquitinase USP36 promotes snoRNP group SUMOylation and is essential for ribosome biogenesis. EMBO Rep. 2021; 22:e50684. 33852194 10.15252/embr.202050684 PMC8183414

[34] Gareau J.R. , Lima C.D. The SUMO pathway: emerging mechanisms that shape specificity, conjugation and recognition. Nat. Rev. Mol. Cell Biol. 2010; 11:861–871. 21102611 10.1038/nrm3011 PMC3079294

[35] Jentsch S. , Psakhye I. Control of nuclear activities by substrate-selective and protein-group SUMOylation. Annu. Rev. Genet. 2013; 47:167–186. 24016193 10.1146/annurev-genet-111212-133453

[36] Moldovan G.L. , Pfander B., Jentsch S. PCNA controls establishment of sister chromatid cohesion during S phase. Mol. Cell. 2006; 23:723–732. 16934511 10.1016/j.molcel.2006.07.007

[37] Desterro J.M. , Rodriguez M.S., Hay R.T. SUMO-1 modification of IkappaBalpha inhibits NF-kappaB activation. Mol. Cell. 1998; 2:233–239. 9734360 10.1016/s1097-2765(00)80133-1

[38] Cubenas-Potts C. , Matunis M.J. SUMO: a multifaceted modifier of chromatin structure and function. Dev. Cell. 2013; 24:1–12. 23328396 10.1016/j.devcel.2012.11.020 PMC3555686

[39] Finkbeiner E. , Haindl M., Raman N., Muller S. SUMO routes ribosome maturation. Nucleus. 2011; 2:527–532. 22064470 10.4161/nucl.2.6.17604

[40] Sarangi P. , Zhao X. SUMO-mediated regulation of DNA damage repair and responses. Trends Biochem. Sci. 2015; 40:233–242. 25778614 10.1016/j.tibs.2015.02.006 PMC4380773

[41] Liu X. , Liu Z., Jang S.W., Ma Z., Shinmura K., Kang S., Dong S., Chen J., Fukasawa K., Ye K. Sumoylation of nucleophosmin/B23 regulates its subcellular localization, mediating cell proliferation and survival. Proc. Natl Acad. Sci. USA. 2007; 104:9679–9684. 17535915 10.1073/pnas.0701806104 PMC1887583

[42] Tago K. , Chiocca S., Sherr C.J. Sumoylation induced by the Arf tumor suppressor: a p53-independent function. Proc. Natl Acad. Sci. USA. 2005; 102:7689–7694. 15897463 10.1073/pnas.0502978102 PMC1129025

[43] Zhang D. , Liang Y., Xie Q., Gao G., Wei J., Huang H., Li J., Gao J., Huang C. A novel post-translational modification of nucleolin, SUMOylation at Lys-294, mediates arsenite-induced cell death by regulating gadd45alpha mRNA stability. J. Biol. Chem. 2015; 290:4784–4800. 25561743 10.1074/jbc.M114.598219 PMC4335216

[44] Castle C.D. , Cassimere E.K., Denicourt C. LAS1L interacts with the mammalian Rix1 complex to regulate ribosome biogenesis. Mol. Biol. Cell. 2012; 23:716–728. 22190735 10.1091/mbc.E11-06-0530 PMC3279398

[45] Finkbeiner E. , Haindl M., Muller S. The SUMO system controls nucleolar partitioning of a novel mammalian ribosome biogenesis complex. EMBO J. 2011; 30:1067–1078. 21326211 10.1038/emboj.2011.33 PMC3061037

[46] Raman N. , Weir E., Muller S. The AAA ATPase MDN1 acts as a SUMO-targeted regulator in mammalian pre-ribosome remodeling. Mol. Cell. 2016; 64:607–615. 27814492 10.1016/j.molcel.2016.09.039

[47] Matic I. , Schimmel J., Hendriks I.A., van Santen M.A., van de Rijke F., van Dam H., Gnad F., Mann M., Vertegaal A.C. Site-specific identification of SUMO-2 targets in cells reveals an inverted SUMOylation motif and a hydrophobic cluster SUMOylation motif. Mol. Cell. 2010; 39:641–652. 20797634 10.1016/j.molcel.2010.07.026

[48] Westman B.J. , Verheggen C., Hutten S., Lam Y.W., Bertrand E., Lamond A.I. A proteomic screen for nucleolar SUMO targets shows SUMOylation modulates the function of Nop5/Nop58. Mol. Cell. 2010; 39:618–631. 20797632 10.1016/j.molcel.2010.07.025 PMC2938476

[49] Hendriks I.A. , Vertegaal A.C. A comprehensive compilation of SUMO proteomics. Nat. Rev. Mol. Cell Biol. 2016; 17:581–595. 27435506 10.1038/nrm.2016.81

[50] Amente S. , Lavadera M.L., Palo G.D., Majello B. SUMO-activating SAE1 transcription is positively regulated by Myc. Am. J. Cancer Res. 2012; 2:330–334. 22679563 PMC3365806

[51] Matafora V. , D’Amato A., Mori S., Blasi F., Bachi A. Proteomics analysis of nucleolar SUMO-1 target proteins upon proteasome inhibition. Mol. Cell. Proteomics. 2009; 8:2243–2255. 19596686 10.1074/mcp.M900079-MCP200 PMC2758753

[52] Vertegaal A.C. , Ogg S.C., Jaffray E., Rodriguez M.S., Hay R.T., Andersen J.S., Mann M., Lamond A.I. A proteomic study of SUMO-2 target proteins. J. Biol. Chem. 2004; 279:33791–33798. 15175327 10.1074/jbc.M404201200

[53] Hendriks I.A. , D'Souza R.C., Yang B., Verlaan-de Vries M., Mann M., Vertegaal A.C Uncovering global SUMOylation signaling networks in a site-specific manner. Nat. Struct. Mol. Biol. 2014; 21:927–936. 25218447 10.1038/nsmb.2890 PMC4259010

[54] Sun X.X. , Chen Y., Su Y., Wang X., Chauhan K.M., Liang J., Daniel C.J., Sears R.C., Dai M.S. SUMO protease SENP1 deSUMOylates and stabilizes c-Myc. Proc. Natl Acad. Sci. USA. 2018; 115:10983–10988. 30305424 10.1073/pnas.1802932115 PMC6205424

[55] Sun X.X. , Challagundla K.B., Dai M.S. Positive regulation of p53 stability and activity by the deubiquitinating enzyme Otubain 1. EMBO J. 2012; 31:576–592. 22124327 10.1038/emboj.2011.434 PMC3273389

[56] Dai M.S. , Lu H. Inhibition of MDM2-mediated p53 ubiquitination and degradation by ribosomal protein L5. J. Biol. Chem. 2004; 279:44475–44482. 15308643 10.1074/jbc.M403722200

[57] Dai M.S. , Arnold H., Sun X.X., Sears R., Lu H. Inhibition of c-Myc activity by ribosomal protein L11. EMBO J. 2007; 26:3332–3345. 17599065 10.1038/sj.emboj.7601776 PMC1933407

[58] Sun X.X. , DeVine T., Challagundla K.B., Dai M.S. Interplay between ribosomal protein S27a and MDM2 protein in p53 activation in response to ribosomal stress. J. Biol. Chem. 2011; 286:22730–22741. 21561866 10.1074/jbc.M111.223651 PMC3123040

[59] Dai M.S. , Zeng S.X., Jin Y., Sun X.X., David L., Lu H. Ribosomal protein L23 activates p53 by inhibiting MDM2 function in response to ribosomal perturbation but not to translation inhibition. Mol. Cell. Biol. 2004; 24:7654–7668. 15314173 10.1128/MCB.24.17.7654-7668.2004 PMC506971

[60] Januszyk K. , Liu Q., Lima C.D. Activities of human RRP6 and structure of the human RRP6 catalytic domain. RNA. 2011; 17:1566–1577. 21705430 10.1261/rna.2763111 PMC3153979

[61] Wasmuth E.V. , Lima C.D. The Rrp6 C-terminal domain binds RNA and activates the nuclear RNA exosome. Nucleic Acids Res. 2017; 45:846–860. 27899565 10.1093/nar/gkw1152 PMC5314766

[62] Bernier-Villamor V. , Sampson D.A., Matunis M.J., Lima C.D. Structural basis for E2-mediated SUMO conjugation revealed by a complex between ubiquitin-conjugating enzyme Ubc9 and RanGAP1. Cell. 2002; 108:345–356. 11853669 10.1016/s0092-8674(02)00630-x

[63] Geiss-Friedlander R. , Melchior F. Concepts in sumoylation: a decade on. Nat. Rev. Mol. Cell Biol. 2007; 8:947–956. 18000527 10.1038/nrm2293

[64] Zhao Q. , Xie Y., Zheng Y., Jiang S., Liu W., Mu W., Liu Z., Zhao Y., Xue Y., Ren J. GPS-SUMO: a tool for the prediction of sumoylation sites and SUMO-interaction motifs. Nucleic Acids Res. 2014; 42:W325–W330. 24880689 10.1093/nar/gku383 PMC4086084

[65] Hendriks I.A. , Lyon D., Young C., Jensen L.J., Vertegaal A.C., Nielsen M.L. Site-specific mapping of the human SUMO proteome reveals co-modification with phosphorylation. Nat. Struct. Mol. Biol. 2017; 24:325–336. 28112733 10.1038/nsmb.3366

[66] Impens F. , Radoshevich L., Cossart P., Ribet D Mapping of SUMO sites and analysis of SUMOylation changes induced by external stimuli. Proc. Natl Acad. Sci. USA. 2014; 111:12432–12437. 25114211 10.1073/pnas.1413825111 PMC4151716

[67] Sloan K.E. , Mattijssen S., Lebaron S., Tollervey D., Pruijn G.J., Watkins N.J. Both endonucleolytic and exonucleolytic cleavage mediate ITS1 removal during human ribosomal RNA processing. J. Cell Biol. 2013; 200:577–588. 23439679 10.1083/jcb.201207131 PMC3587827

[68] Ashcroft M. , Taya Y., Vousden K.H. Stress signals utilize multiple pathways to stabilize p53. Mol. Cell. Biol. 2000; 20:3224–3233. 10757806 10.1128/mcb.20.9.3224-3233.2000 PMC85616

[69] Drygin D. , Lin A., Bliesath J., Ho C.B., O’Brien S.E., Proffitt C., Omori M., Haddach M., Schwaebe M.K., Siddiqui-Jain A. et al. Targeting RNA polymerase I with an oral small molecule CX-5461 inhibits ribosomal RNA synthesis and solid tumor growth. Cancer Res. 2011; 71:1418–1430. 21159662 10.1158/0008-5472.CAN-10-1728

[70] Petit F.G. , Jamin S.P., Kernanec P.Y., Becker E., Halet G., Primig M. 2021) EXOSC10/Rrp6 is essential for the eight-cell embryo/morula transition. Dev. Biol. 483:58–65. 34965385 10.1016/j.ydbio.2021.12.010

[71] Psakhye I. , Jentsch S. Protein group modification and synergy in the SUMO pathway as exemplified in DNA repair. Cell. 2012; 151:807–820. 23122649 10.1016/j.cell.2012.10.021

[72] Hendriks I.A. , D'Souza R.C., Chang J.G., Mann M., Vertegaal A.C System-wide identification of wild-type SUMO-2 conjugation sites. Nat. Commun. 2015; 6:7289. 26073453 10.1038/ncomms8289 PMC4490555

[73] Hendriks I.A. , Treffers L.W., Verlaan-de Vries M., Olsen J.V., Vertegaal A.C SUMO-2 orchestrates chromatin modifiers in response to DNA damage. Cell Rep. 2015; 10:1778–1791. 25772364 10.1016/j.celrep.2015.02.033 PMC4514456

[74] Lamoliatte F. , Caron D., Durette C., Mahrouche L., Maroui M.A., Caron-Lizotte O., Bonneil E., Chelbi-Alix M.K., Thibault P. Large-scale analysis of lysine SUMOylation by SUMO remnant immunoaffinity profiling. Nat. Commun. 2014; 5:5409. 25391492 10.1038/ncomms6409

[75] Tammsalu T. , Matic I., Jaffray E.G., Ibrahim A.F.M., Tatham M.H., Hay R.T. Proteome-wide identification of SUMO2 modification sites. Sci. Signal. 2014; 7:rs2. 24782567 10.1126/scisignal.2005146 PMC4051997

[76] Xiao Z. , Chang J.G., Hendriks I.A., Sigurethsson J.O., Olsen J.V., Vertegaal A.C. System-wide analysis of SUMOylation dynamics in response to replication stress reveals novel small ubiquitin-like modified target proteins and acceptor lysines relevant for genome stability. Mol. Cell. Proteomics. 2015; 14:1419–1434. 25755297 10.1074/mcp.O114.044792 PMC4424410

[77] Knight J.R. , Bastide A., Peretti D., Roobol A., Roobol J., Mallucci G.R., Smales C.M., Willis A.E. Cooling-induced SUMOylation of EXOSC10 down-regulates ribosome biogenesis. RNA. 2016; 22:623–635. 26857222 10.1261/rna.054411.115 PMC4793216

[78] Treiber T. , Treiber N., Plessmann U., Harlander S., Daiss J.L., Eichner N., Lehmann G., Schall K., Urlaub H., Meister G. A compendium of RNA-binding proteins that regulate microRNA biogenesis. Mol. Cell. 2017; 66:270–284. 28431233 10.1016/j.molcel.2017.03.014

