# Supplementary material for: The ubiquitin-specific protease USP36 SUMOylates EXOSC10 and promotes the nucleolar RNA exosome function in rRNA processing
Source: Nucleic Acids Res. 2023 Mar 13;51(8):3934–49. doi: 10.1093/nar/gkad140 (PMC10164564; doi:10.1093/nar/gkad140)
Supplement: gkad140_Supplemental_Files [file gkad140_supplemental_files.zip › Chen-USP36_exosome_ Supplementary.pdf]

Supplementary information

The Ubiquitin-Specific Protease USP36 SUMOylates EXOSC10 and promotes the nucleolar RNA exosome function in rRNA processing

Yingxiao Chen[1], Yanping Li[1], Roselyn S. Dai[1], Jonathan C. Savage[2], Ujwal Shinde[2], John Klimek[3], Larry L. David[2,3], Emma A. Young[4], Markus Hafner[4], Rosalie C. Sears[1], Xiao-Xin Sun[1*], Mu-Shui Dai[1*]

Supplementary Figure S1

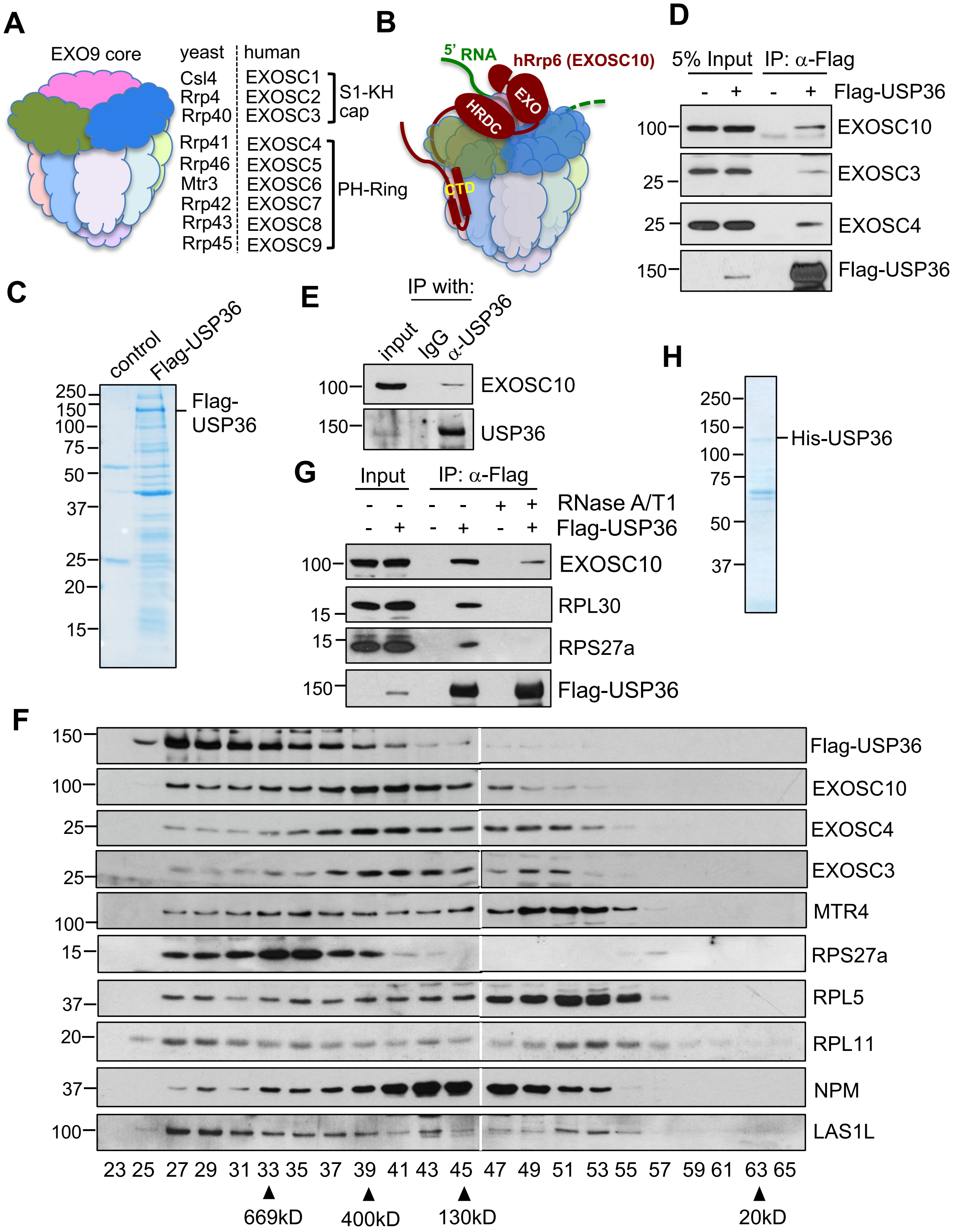

Supplementary Figure S1. USP36 interacts with the RNA exosome in cells.

(A). Schematic diagram of the 9-subunit RNA exosome core. The yeast genes and their human orthologs are listed on the left and right, respectively. (**B**). Schematic diagram of the nucleolar EXO10^{EXOSC10}. EXO, exonuclease domain; HRDC: helicase and RNase D C-terminal domain. CTD, C-terminal domain. **(C).** Colloidal blue staining of SDS-PAGE gel of affinity purified Flag-USP36-associated protein complex compared to control vector expressing 293 cells. Flag-USP36 band is indicated. **(D).** USP36 interacts with the RNA exosome. HeLa cells transfected with Flag-USP36 or control vector were subjected to co-IP with anti-flag antibody followed by IB. **(E).** Endogenous UP36 interacts with endogenous EXOSC10. HeLa cell lysates were immunoprecipitated with anti-USP36 antibody (Proteintech) followed by IB with anti-EXOSC10. **(F).** Detection of Flag-USP36, exosome components, MTR4 adaptor proteins, NPM, Las1L and ribosomal proteins in fractions eluted from Superose 6 column loaded with 293-Flag-USP36 cell lysates. Molecular weight markers are indicated at the bottom. **(G).** Interaction between USP36 and EXOSC10 is partially dependent on RNA. H1299 cells transfected with Flag-USP36 were subjected to co-IP with anti-flag antibody in the presence or absence of 100 μg/mL RNase A and 100U/ml RNase T1 followed by IB. **(H).** Coomassie staining showing the purified His-USP36 recombinant protein used for GST-pull down assays in Figure 1I.

Supplementary Figure S2

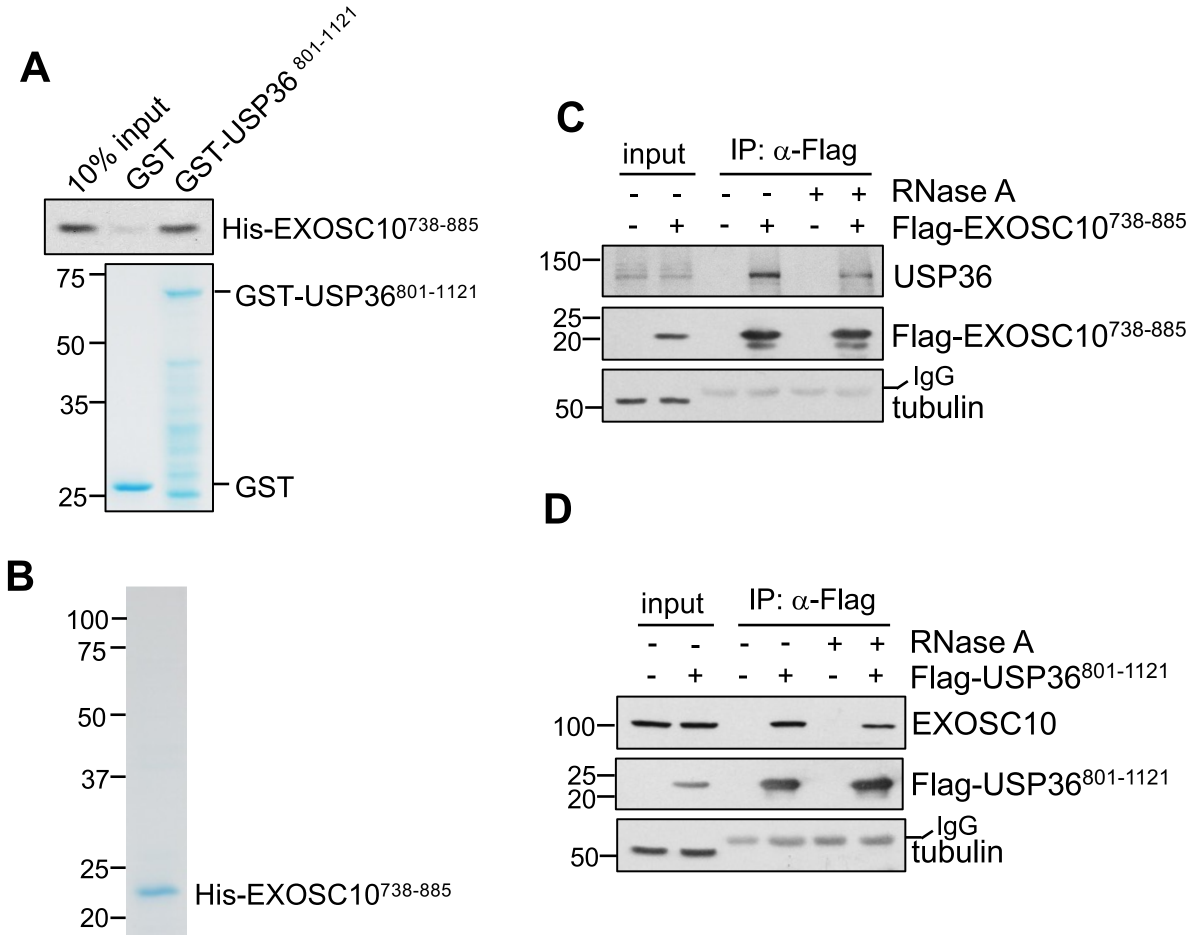

Supplementary Figure S2. Interaction of USP36 and EXOSC10 via their C-terminal domains. (A). The C-terminus of USP36 directly interacts with C-terminal Lasso domain of EXOSC10 *in vitro*. Purified GST or GST-USP36$^{801\text{-}1121}$ immobilized on glutathione beads was incubated with purified His-EXOSC10$^{738\text{-}885}$. Bound proteins were assayed by IB with anti-EXOSC10 (top). Coomassie staining of GST and GST-USP36$^{801\text{-}1121}$ proteins is shown at the bottom panel. **(B).** His-EXOSC10$^{738\text{-}885}$ recombinant protein purified from bacteria and used in above (A) was stained by Coomassie staining. **(C) (D).** Interaction between USP36 and EXOSC10 C-termini is partially dependent on RNA. H1299 cells transfected with Flag- EXOSC10$^{738\text{-}885}$ (C) or USP36$^{801\text{-}1121}$ (D) were subjected to co-IP with anti-flag antibody in the presence or absence of RNase A followed by IB.

Supplementary Figure S3

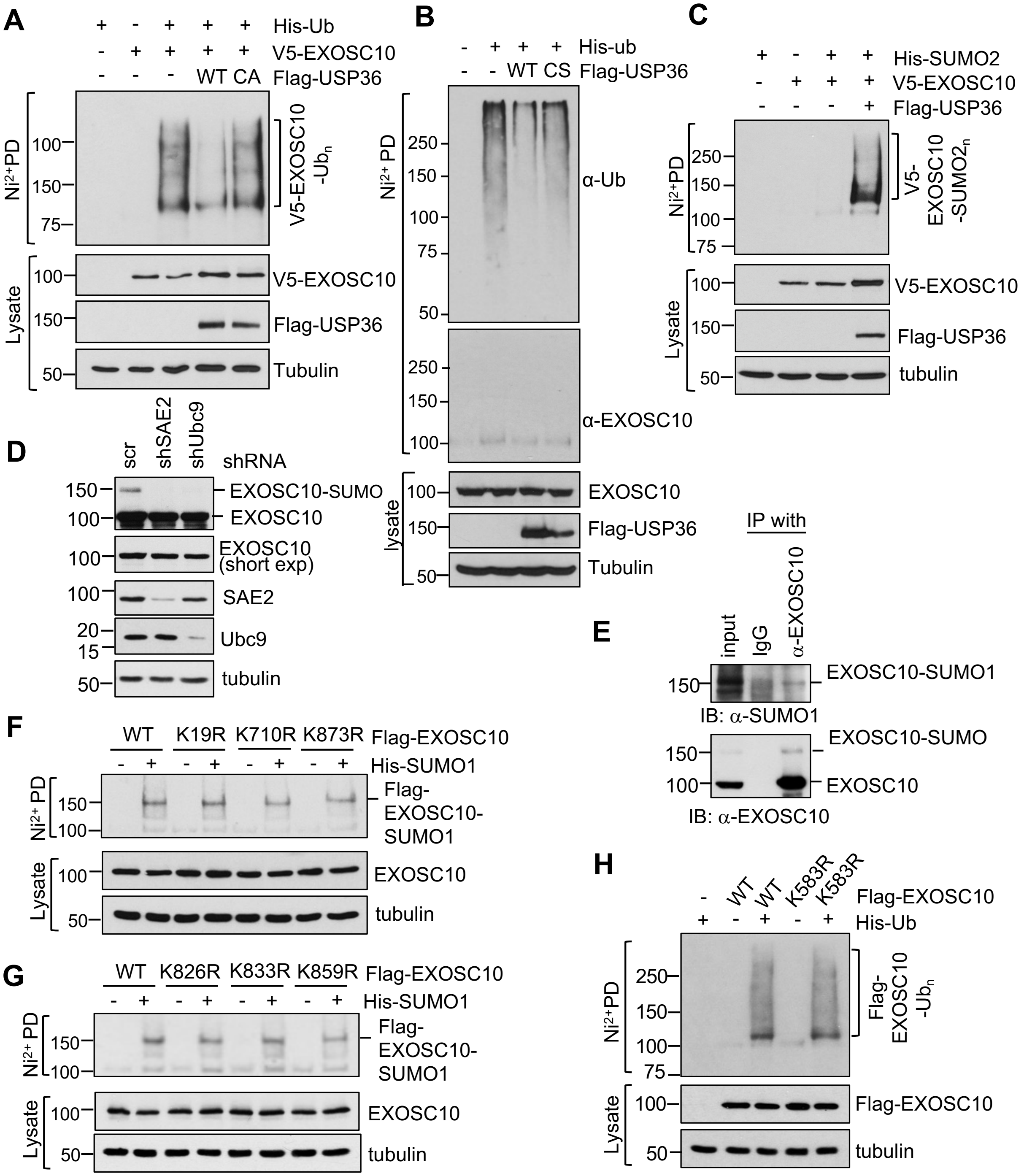

Supplementary Figure S3. Effects of USP36 on EXOSC10 ubiquitination and SUMOylation. (A). Overexpression of USP36 deubiquitinates exogenously expressed EXOSC10. H1299 cells were transfected with His-Ub, V5-EXOSC10 together with or without Flag-USP36 (WT or its catalytically inactive C131A mutant) plasmids and treated with MG132 (40 μM) for 6 hours before harvesting. The cells were then subjected to Ni^{2+}-NTA PD under denature conditions, followed by IB. The protein expression is shown at the bottom panels. **(B).** Endogenous EXOSC10 ubiquitination is under detectable. H1299 cells transfected with His-Ub with or without WT Flag-USP36 or its catalytically inactive C131A mutant and treated with MG132 (40 μM) for 6 hours before harvesting. The cells were then subjected to Ni^{2+}-NTA PD under denature conditions, followed by IB. The protein expression is shown at the bottom panels. **(C).** USP36 promotes SUMO2 modification of EXOSC10. H1299 cells transfected with the indicated plasmids were subjected to Ni^{2+}-NTA PD followed by IB to detect EXOSC10 SUMOylation. The SUMO2 modified EXOSC10 is indicated. The protein expression is shown at the bottom panels. **(D).** Knockdown of SAE2 or Ubc9 attenuates the SUMOylation of EXOSC10. HeLa cells infected with lentiviruses encoding SAE2 or Ubc9 shRNA were assayed by IB. **(E).** Confirmation of the modified EXOSC10 form as SUMOylated EXOSC10. HeLa cells were immunoprecipitated with anti-EXOSC10 antibody followed by IB with anti-SUMO1 antibody. **(F)(G).** Mutating K19, K710, K826, K833, K859 or K873 to Arg does not abolish EXOSC10 SUMOylation. H1299 cells transfected with the indicated plasmids were subjected to Ni^{2+}-NTA PD under denaturing conditions followed by IB. **(H).** Mutating K583 to Arg does not affect EXOSC10 ubiquitination. H1299 cells were transfected with indicated plasmids and treated with MG132 (40 μM) for 6 hours before harvesting. The cells were then subjected to Ni^{2+}-NTA PD under denature conditions, followed by IB. The protein expression is shown at the bottom panels.

Supplementary Figure S4

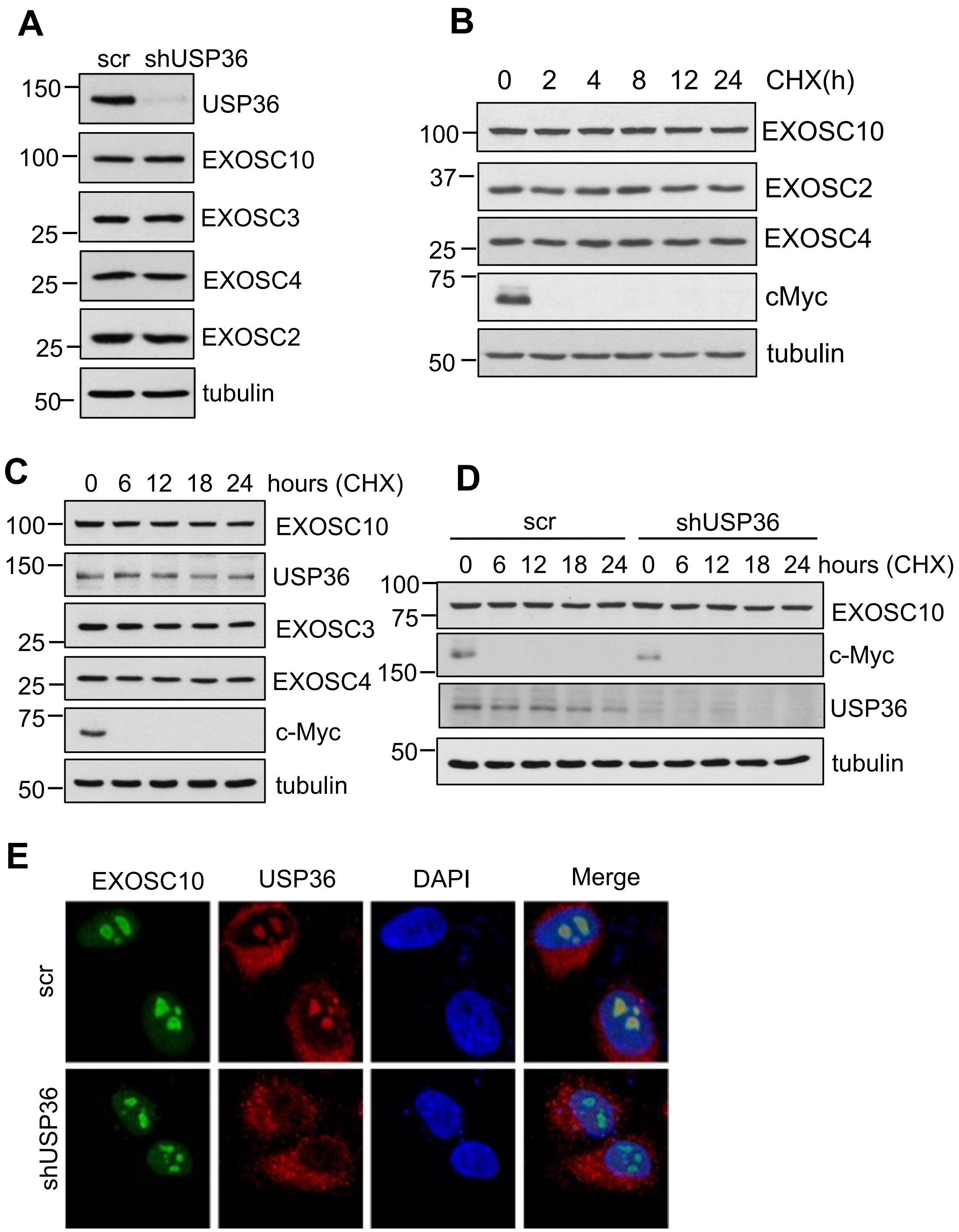

Supplementary Figure S4. USP36 does not affect EXOSC10 levels and nucleolar localization.

(A). USP36 does not affect the levels of exosome proteins. HeLa cells infected with scr or shUSP36 lentivirus were assayed by IB. **(B) (C).** Measurement of the half-life of the RNA exosome proteins. HeLa (B) or H1299 (C) cells were treated with 100 μg/mL cycloheximide (CHX) and harvested at different time points followed by IB detection of the indicated proteins. **(D)** USP36 does not affect the half-life of EXOSC10. HeLa cells infected with the indicated shRNA lentiviruses were treated with 100 μg/mL cycloheximide (CHX) and harvested at different time points followed by IB. c-Myc served as a positive control. **(E)** USP36 does not affect the nucleolar localization of EXOSC10. HeLa cells infected with scr or shUSP36 lentiviruses were immunostained with anti-USP36 (Red) and anti-EXOSC10 (Green). The nuclei were stained with DAPI (blue).

Supplementary Figure S5

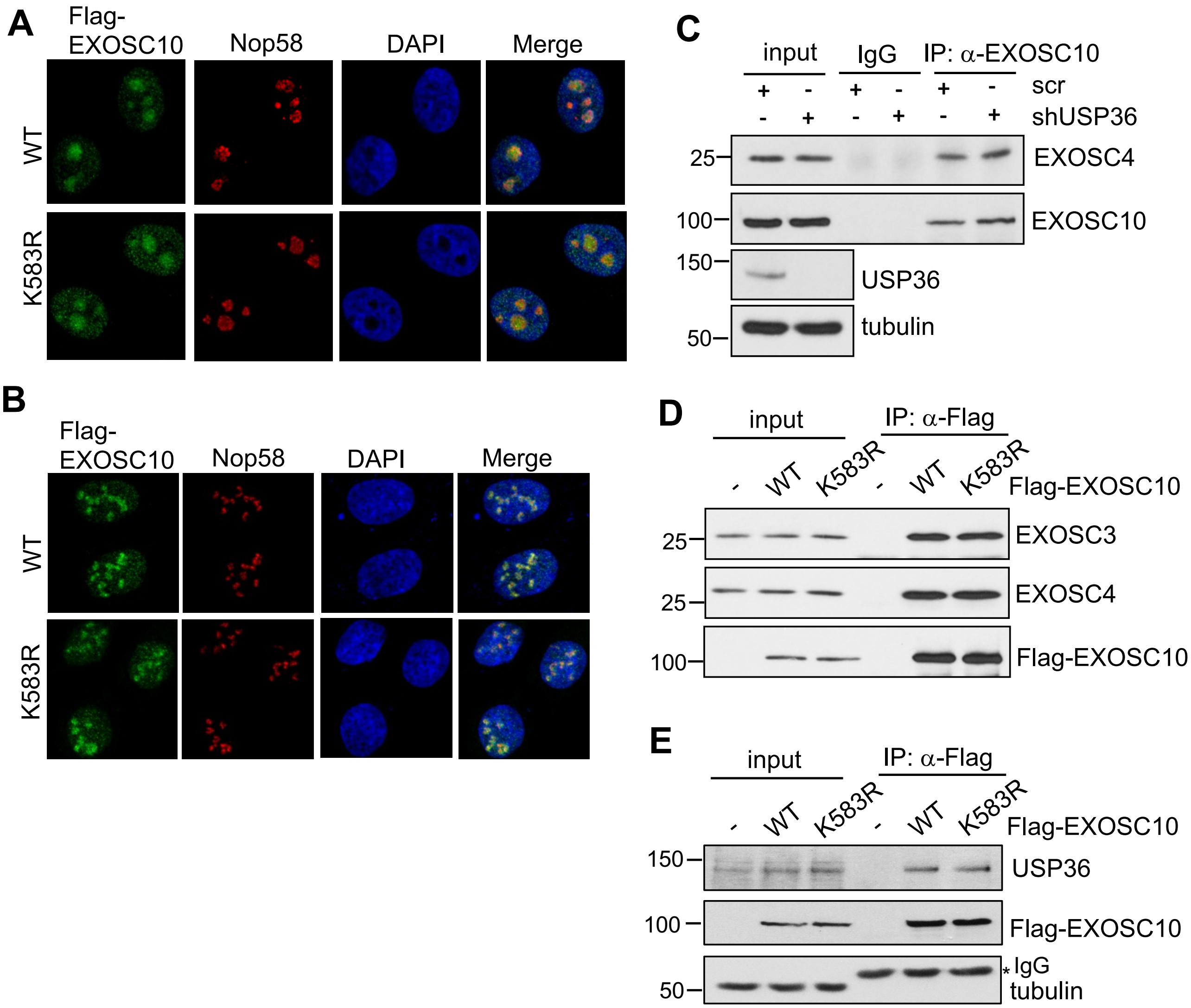

Supplementary Figure S5. USP36 does not affect EXOSC10 levels and nucleolar localization.

(A) (B). EXOSC10 SUMOylation at K583 does not affect its nucleolar localization. HeLa (A) and U2OS (B) cells transfected with Flag-EXOSC10 (WT or the K583R mutant) were immunostained with anti-Flag (Green) and the nucleolar protein Nop58 (Red). The nuclei were stained with DAPI (blue). **(C).** USP36 knockdown does not affect the association between EXOSC10 and EXOSC4. HeLa cells infected with scr or shUSP36 lentiviruses were subjected to co-IP with anti-EXOSC10 followed by IB. **(D).** K583R mutation of EXOSC10 does not affect EXOSC10 association with core exosome components. H1299 cells transfected with the indicated plasmids were assayed by co-IP with anti-Flag followed by IB. **(E).** K583R mutation of EXOSC10 does not affect EXOSC10 association with USP36. H1299 cells transfected with the indicated plasmids were assayed by co-IP with anti-Flag followed by IB.

Supplementary Figure S6

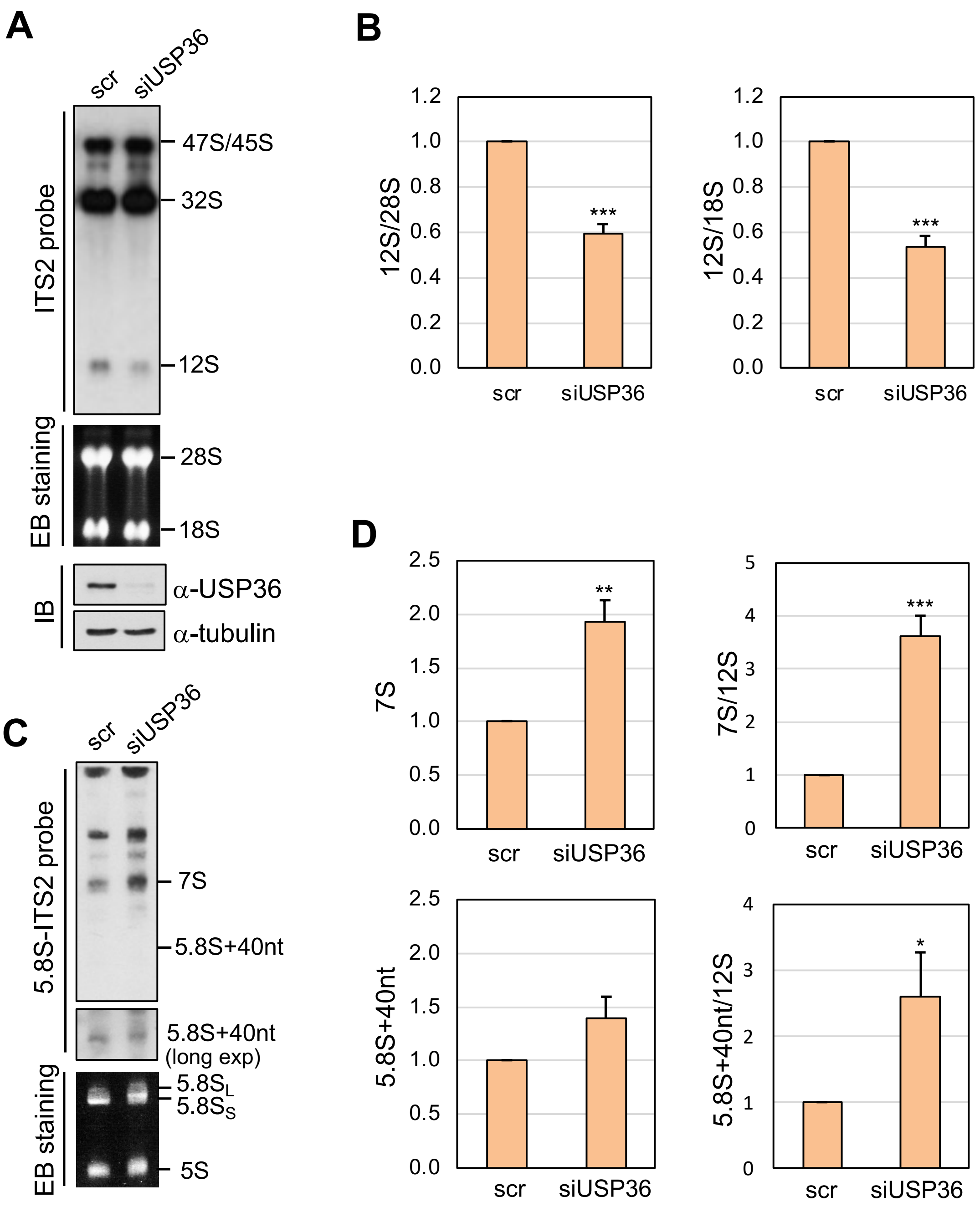

Supplementary Figure S6. Knockdown of USP36 inhibits rRNA processing.

(A) (B). Knockdown of USP36 reduces the levels of 12S rRNA. HeLa cells transfected with scr or USP36 siRNAs were assayed for rRNA processing by Northern blot using ITS2 probe. EB staining of the RNA agarose gel is shown in middle panel and IB showing the knockdown of USP36 is shown in the bottom panels (A). The 12S rRNA species were quantified and the ratios of 12S rRNA to 28S and 18S rRNA were calculated from four independent experiments (B). ***, $P<0.001$, comparison between scr and USP36 siRNA transfected cells as determined by Student's t-test.

(C) (D). Knockdown of USP36 impairs 12S rRNA processing. HeLa cells transfected with scr or USP36 siRNAs were assayed for rRNA processing by Northern blot using 5.8S-ITS2 junction probe. EB staining of the RNA gel is shown in bottom panel (C). The 7S and 5.8S+40nt species were quantified and the ratios of 7S and 5.8S+40nt species to 12S rRNA were calculated from four independent experiments (D). *, $P<0.05$, **, $P<0.01$, ***, $P<0.001$, compared to scr RNA transfected cells as determined by Student's t-test.

Supplementary Figure S7

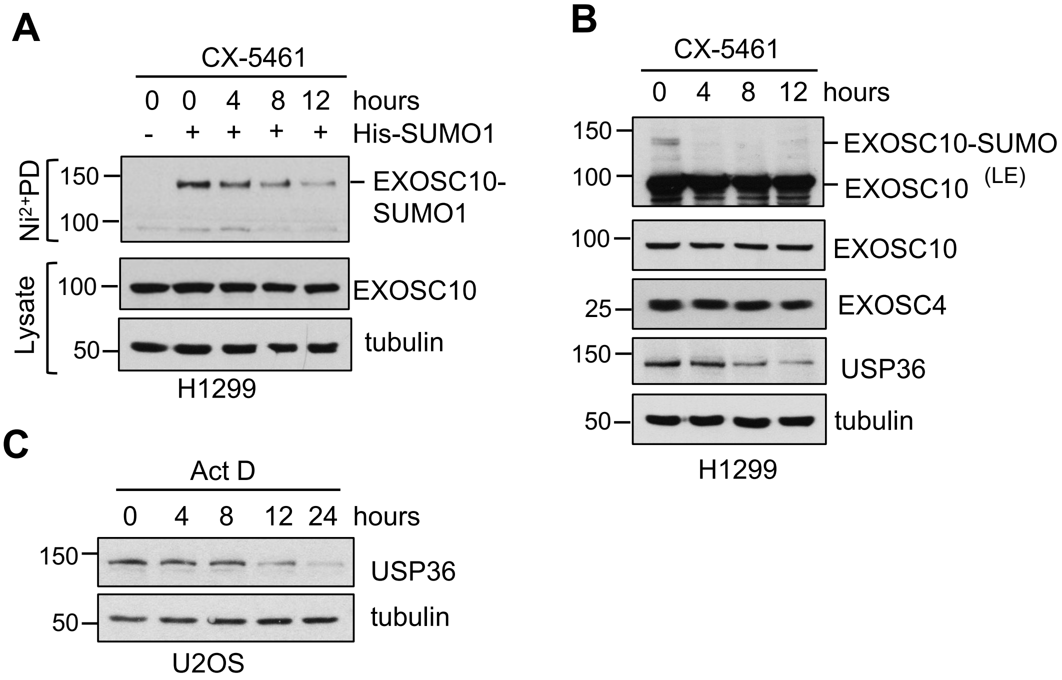

Supplementary Figure S7. EXOSC10 SUMOylation and USP36 expression are reduced in cells in response to the perturbation of ribosome biogenesis.

(A) EXOSC10 SUMOylation is reduced in cells by the treatment with CX-5461. H1299 cells transfected with or without His-SUMO1 were treated with or without 1 μM CX-5461 for different times, followed by Ni^{2+}-NTA PD under denaturing conditions to detect SUMOylated EXOSC10 by IB**.** The protein expression is shown at the bottom panels. **(B)** EXOSC10 modified by endogenous SUMO is reduced in cells in response to the treatment of CX-5461. H1299 cells were treated with or without 1 μM CX-5461 for different times, followed by IB**.** **(C)** USP36 is reduced in cells by the a low dose of Act D treatment. U2OS cells treated with 5 nM Act D for different times were analyzed by IB.

Supplementary Table 1: Top 20 Flag-USP36 interacting proteins*

			Spectral Counts	
Accession	Gene	Protein name	Flag-USP36	Control
Q9H6R0	DHX33	ATP-dependent RNA helicase DHX33	117	0
Q9UNX4	WDR3	WD repeat-containing protein 3	82	0
Q12788	TBL3	Transducin beta-like protein 3	71	0
Q14690	RRP5	Protein RRP5 homolog	62	0
O00567	NOP56	Nucleolar protein 56	54	0
Q8NI36	WDR36	WD repeat-containing protein 36	52	0
Q9H0A0	NAT10	RNA cytidine acetyltransferase	52	0
Q8IY81	SPB1	pre-rRNA 2'-O-ribose RNA methyltransferase FTSJ3	48	0
Q9H583	HEAT1	HEAT repeat-containing protein 1	45	0
Q9Y2X3	NOP58	Nucleolar protein 58	45	0
Q9Y5J1	UTP18	U3 small nucleolar RNA-associated protein 18 homolog	41	0
Q9NW13	RBM28	RNA-binding protein 28	38	0
Q96GQ7	DDX27	Probable ATP-dependent RNA helicase DDX27	37	0
Q9P2E9	RRBP1	Ribosome-binding protein 1	37	0
Q8WTT2	NOC3L	Nucleolar complex protein 3 homolog	35	0
O00541	PESC	Pescadillo homolog	33	0
Q14974	IMB1	Importin subunit beta-1	33	0
Q01780	EXOSC10	Exosome component 10	31	0
Q14137	BOP1	Ribosome biogenesis protein BOP1	31	0
Q9H6R4	NOL6	Nucleolar protein 6	31	0

* Proteins with the top 20 greatest number of assigned MS/MS spectra (spectral counts) in the Flag-USP36 sample that also had 0 spectral counts in the control sample.
