# Supplementary material for: The ubiquitin-specific protease USP36 SUMOylates EXOSC10 and promotes the nucleolar RNA exosome function in rRNA processing
Source: Nucleic Acids Res. 2023 Mar 13;51(8):3934–49. doi: 10.1093/nar/gkad140 (PMC10164564; doi:10.1093/nar/gkad140)

Source Images for Supplementary Figure 1

Figure S1C

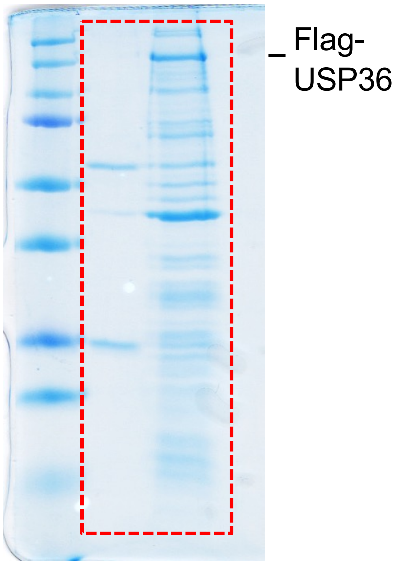

Figure S1D

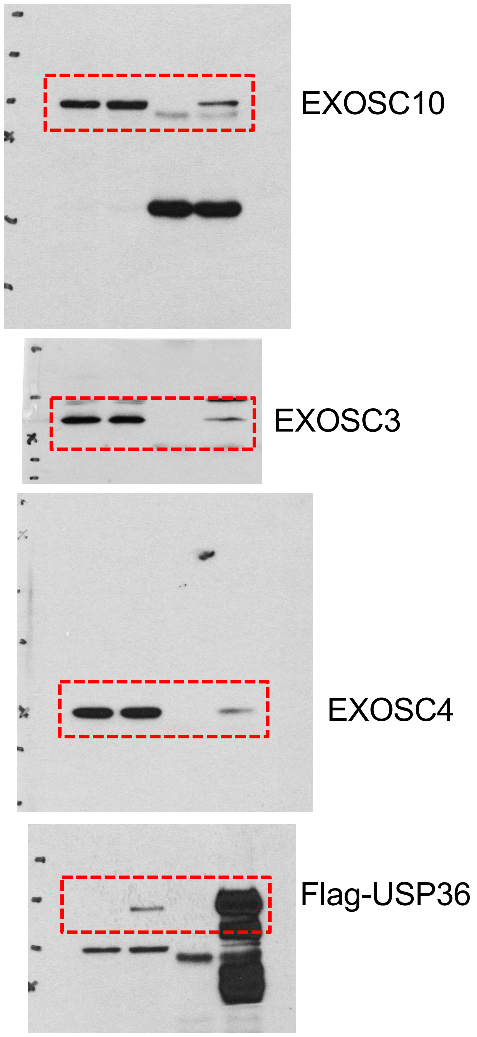

Figure S1E

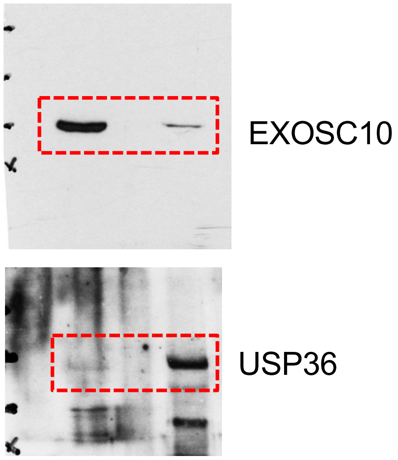

Figure S1G

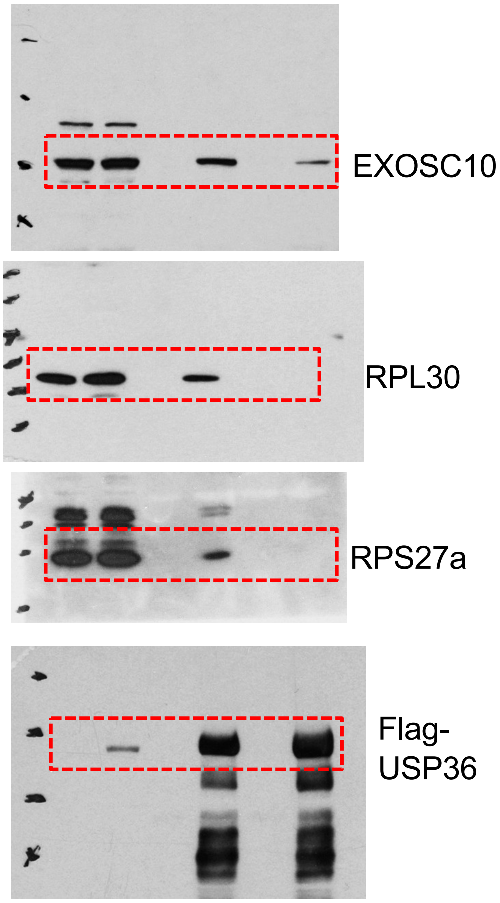

Figure S1H

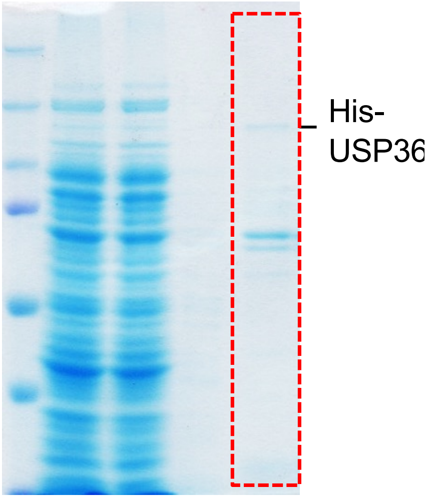

Source Images for Supplementary Figure 1

Figure S1F

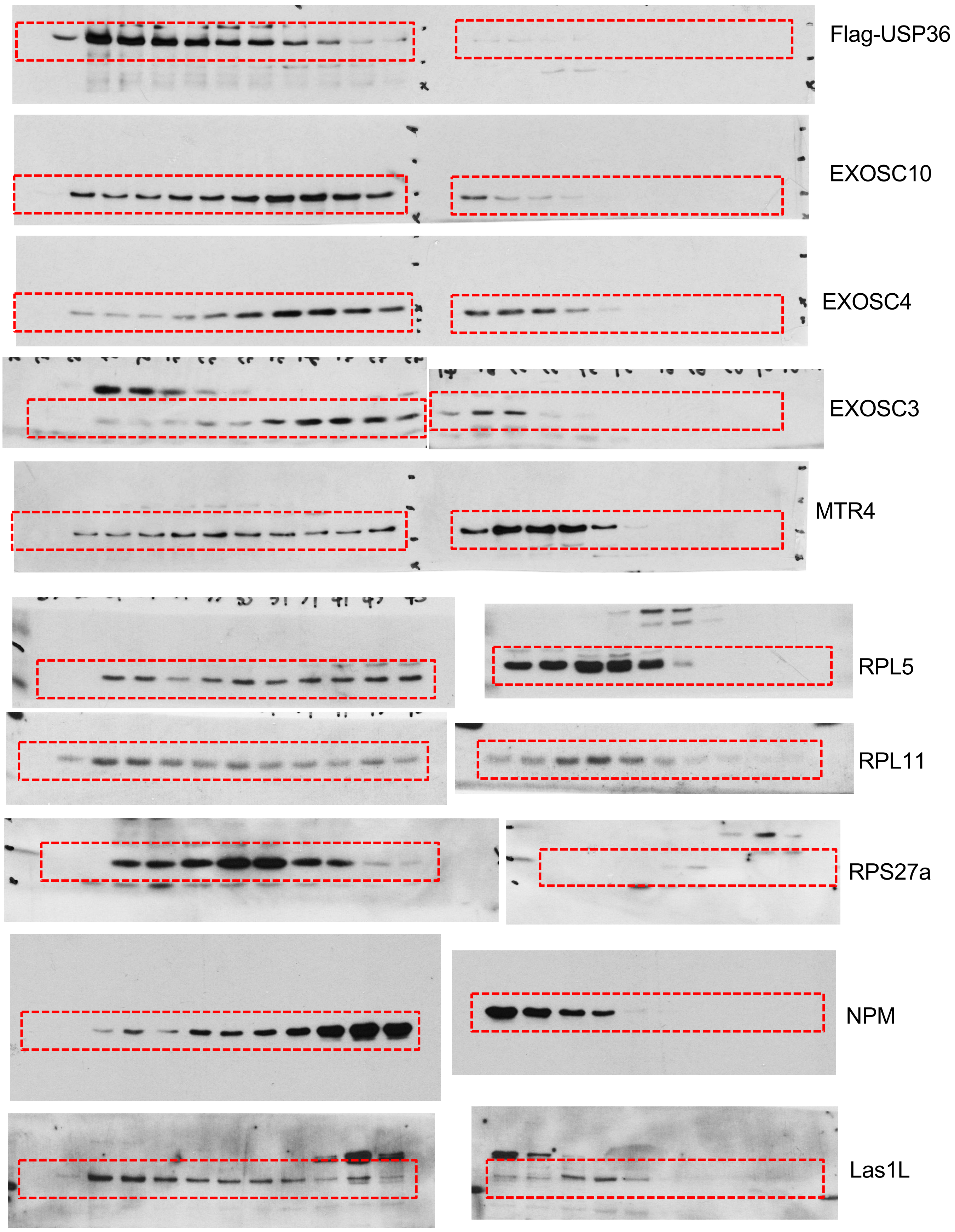

Source Images for Supplementary Figure 2

Figure S2A, S2B

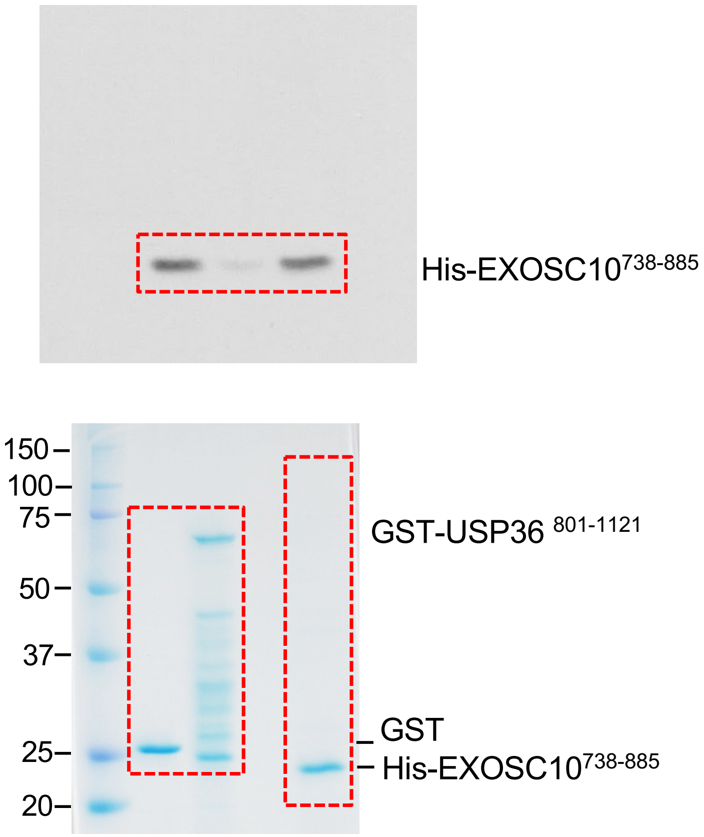

Figure S2C

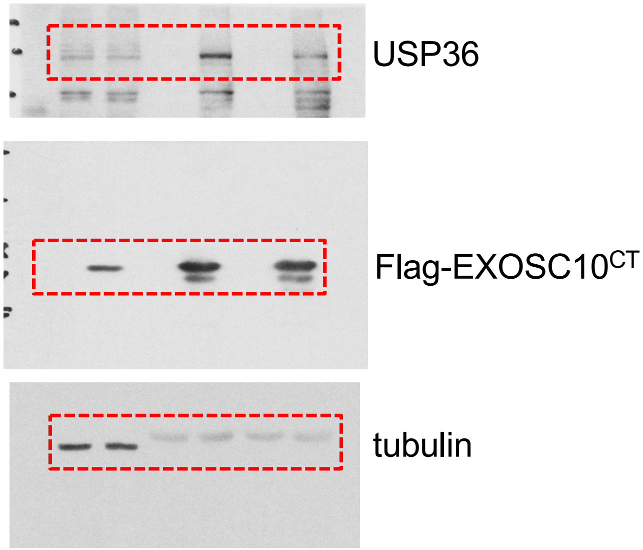

Figure S2D

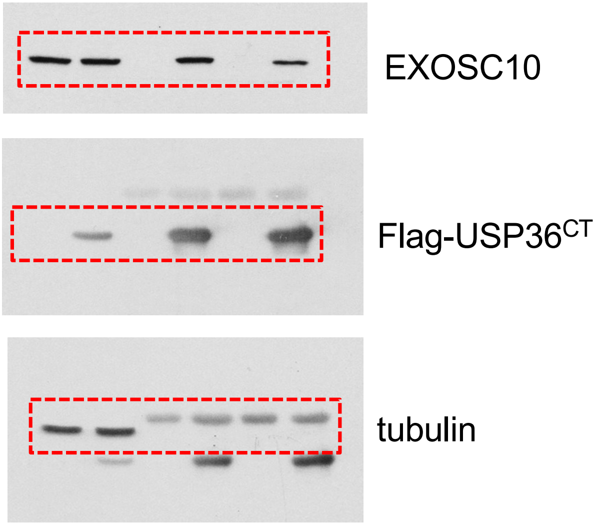

Source Images for Supplementary Figure 3

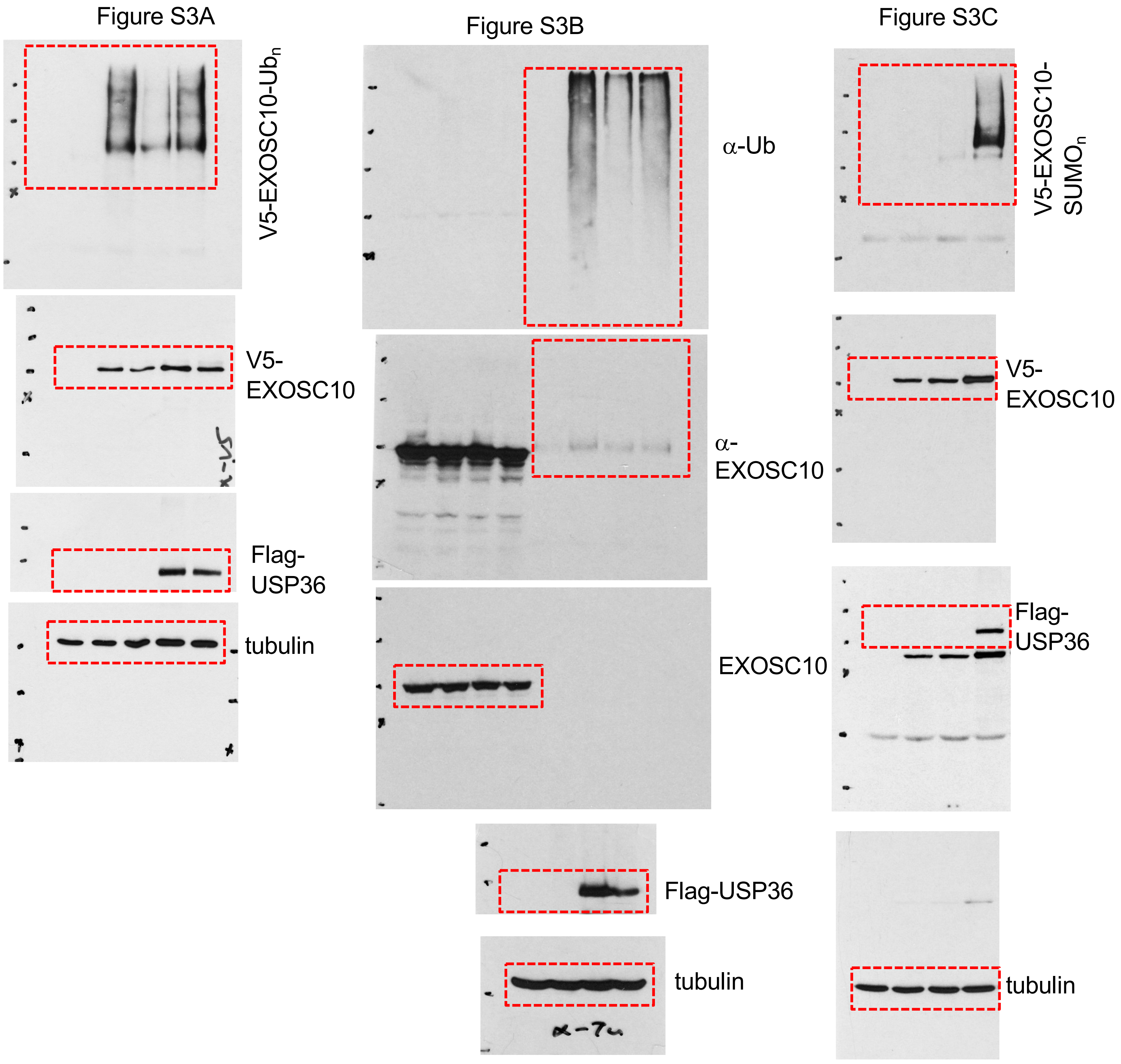

Source Images for Supplementary Figure 3

Figure S3D

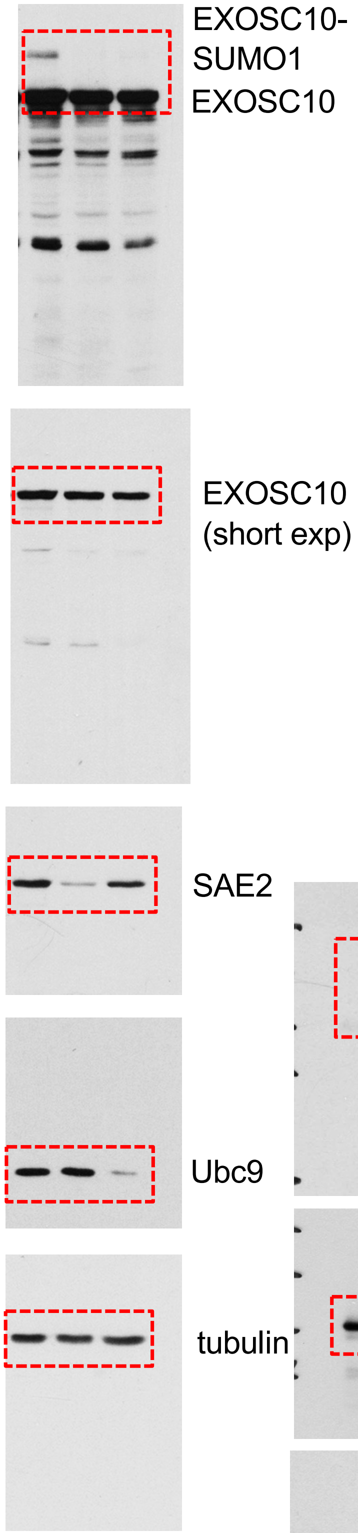

Figure S3E

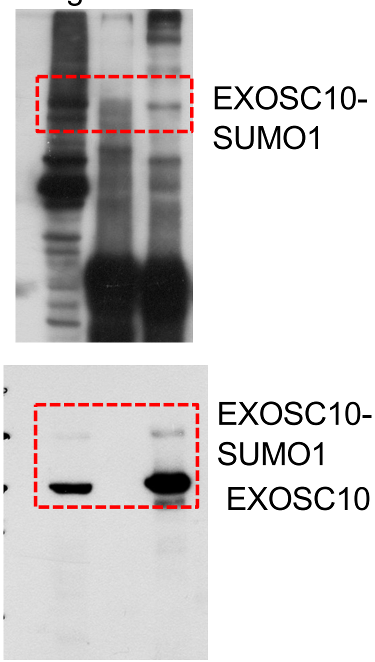

Figure S3F

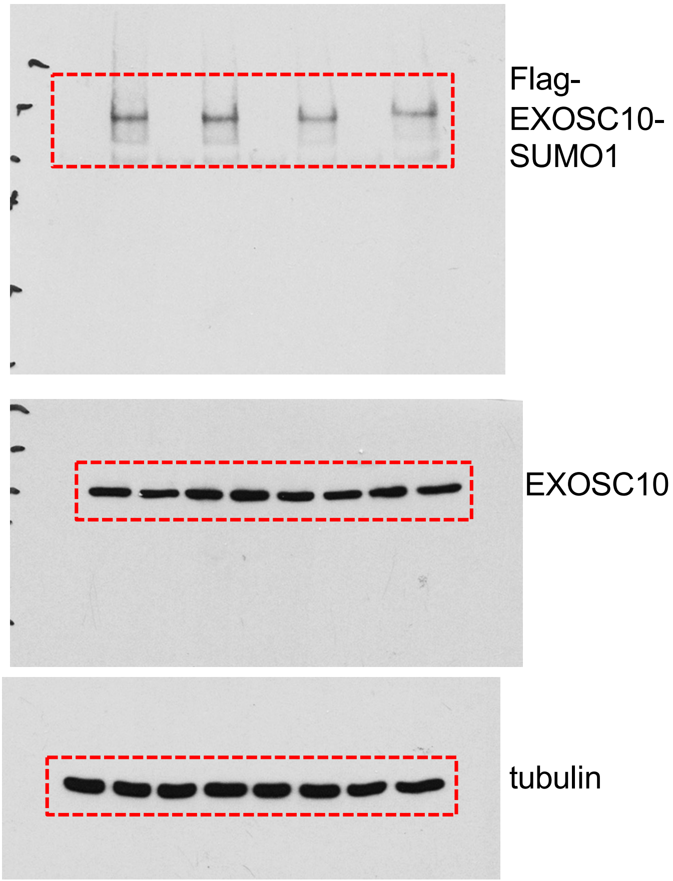

Figure S3G

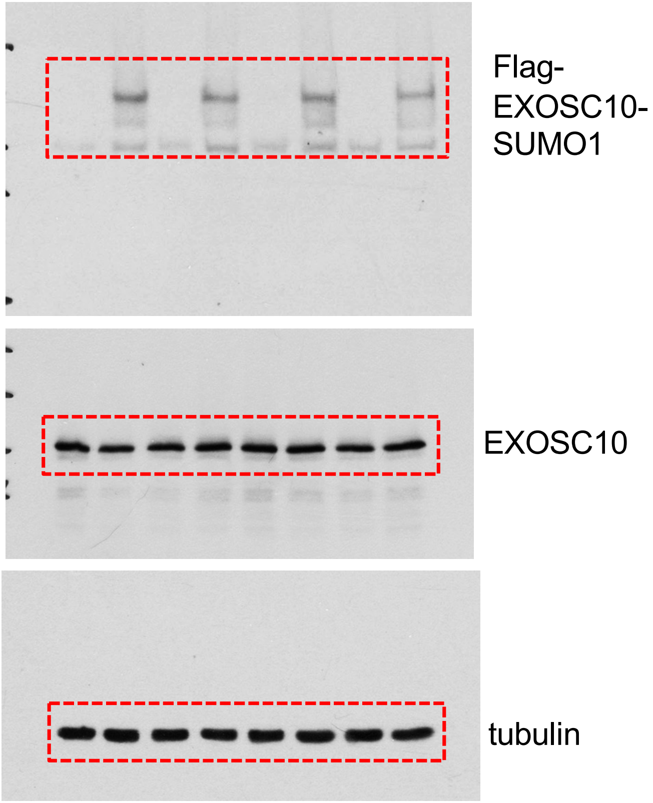

Figure S3H

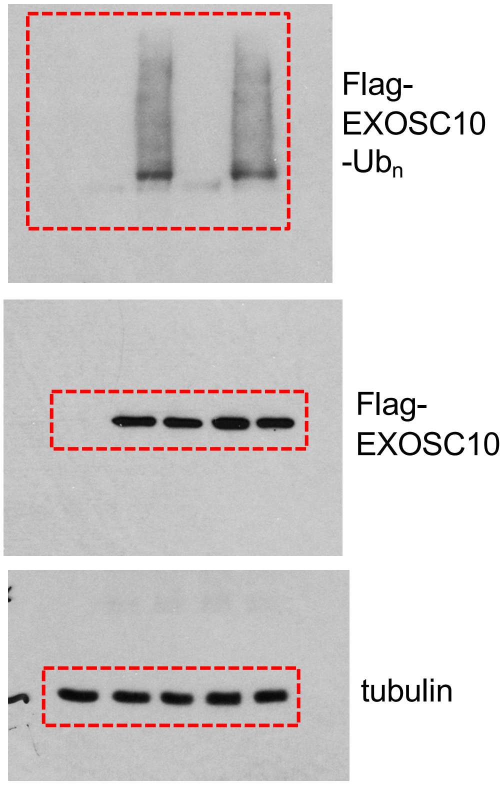

Source Images for Supplementary Figure 4

Figure S4A

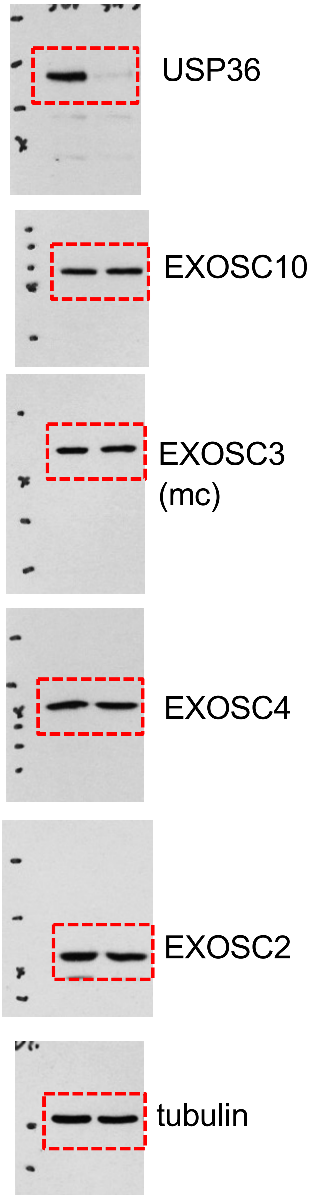

Figure S4B

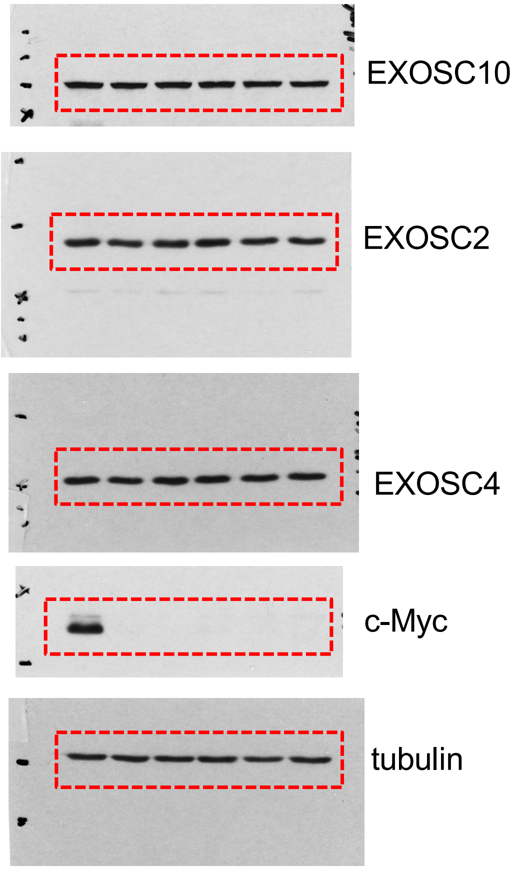

Figure S4C

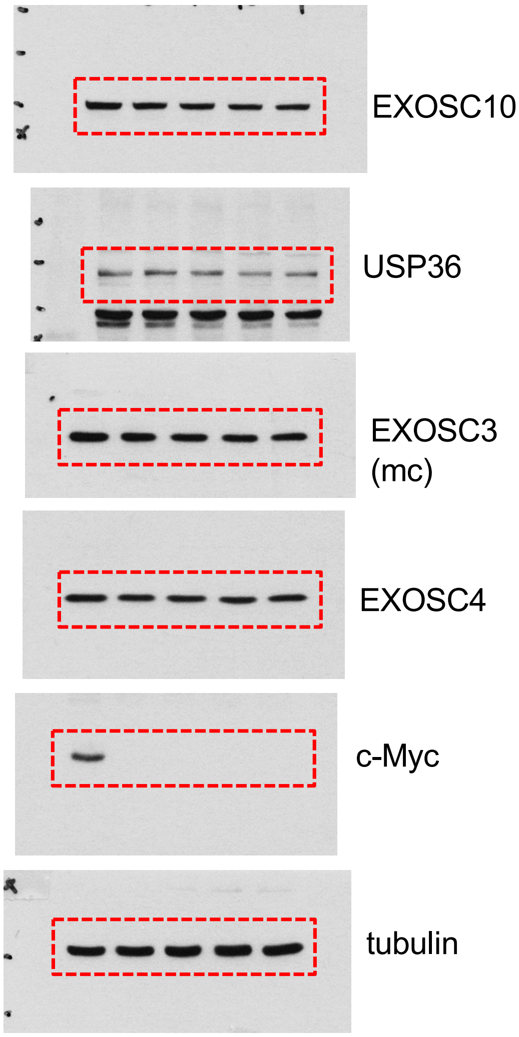

Figure S4D

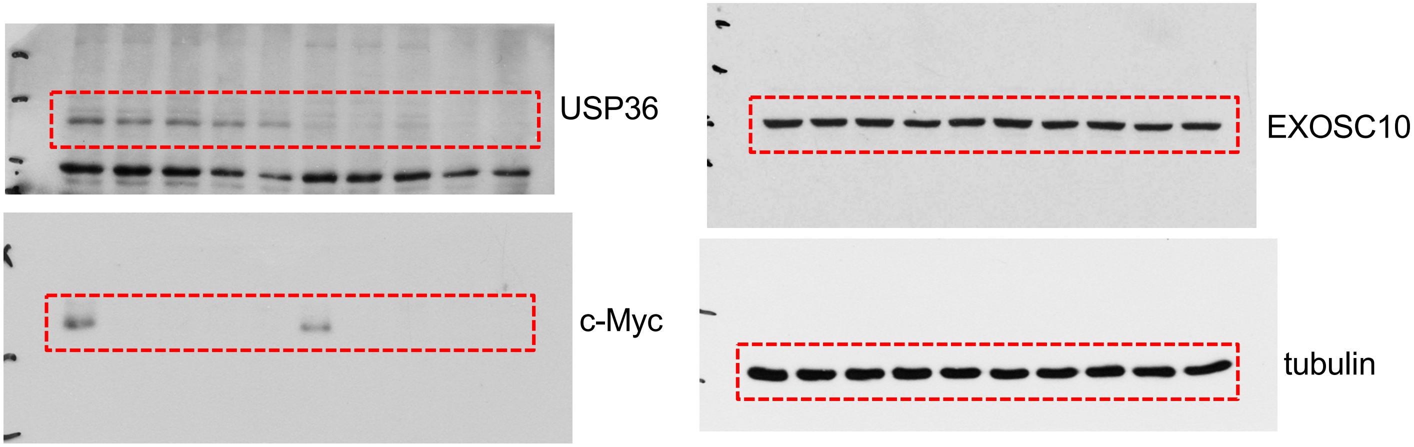

Source Images for Supplementary Figure 5

Figure S5C

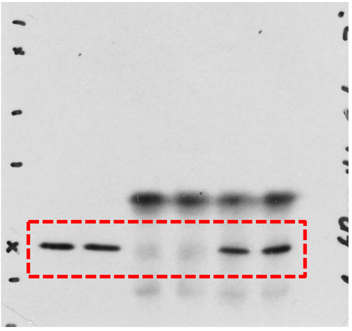
EXOSC4

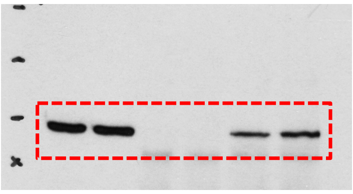
EXOSC10

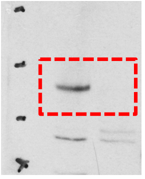
USP36

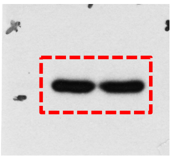
tubulin

Figure S5D

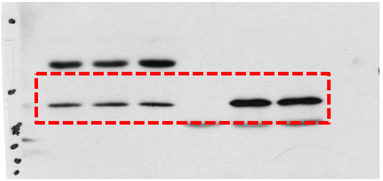
EXOSC3

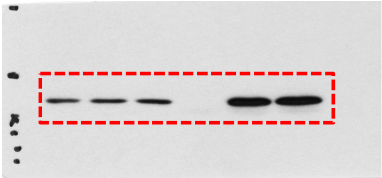
EXOSC4

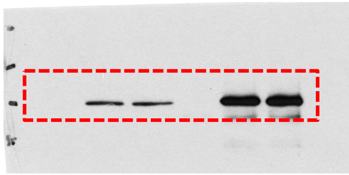
Flag-EXOSC10

Figure S5E

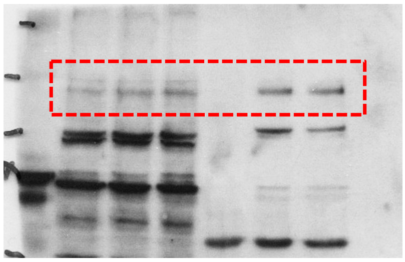
USP36

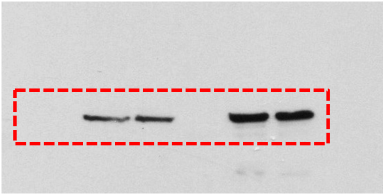
Flag-EXOSC10

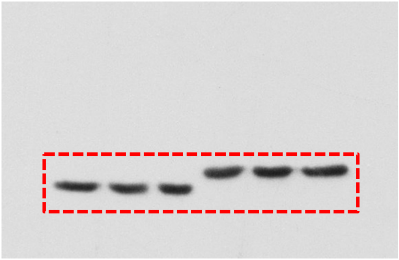
tubulin

Source Images for Supplementary Figure 6

Figure S6A

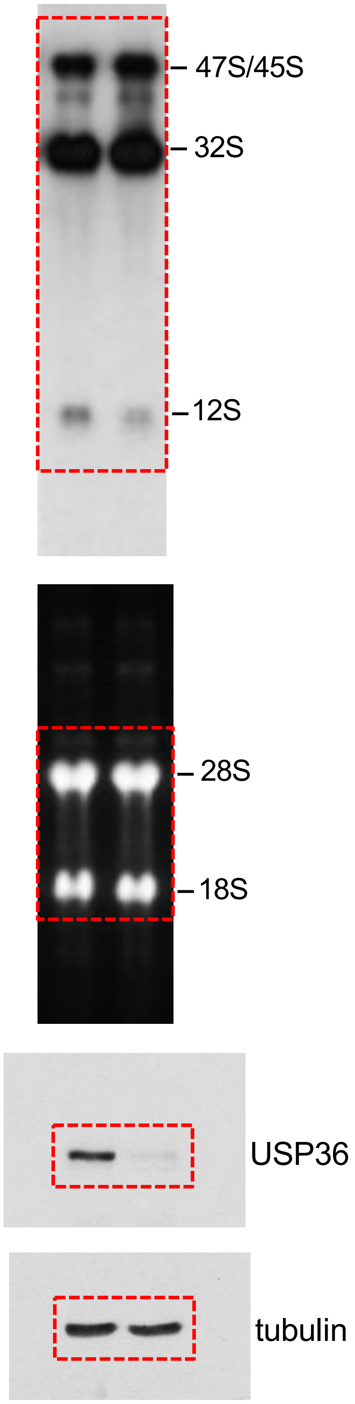

Figure S6B

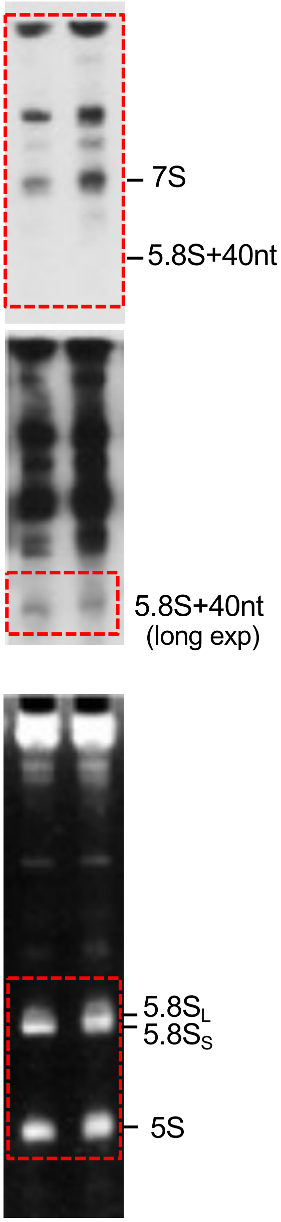

Source Images for Supplementary Figure 7

Figure S7A

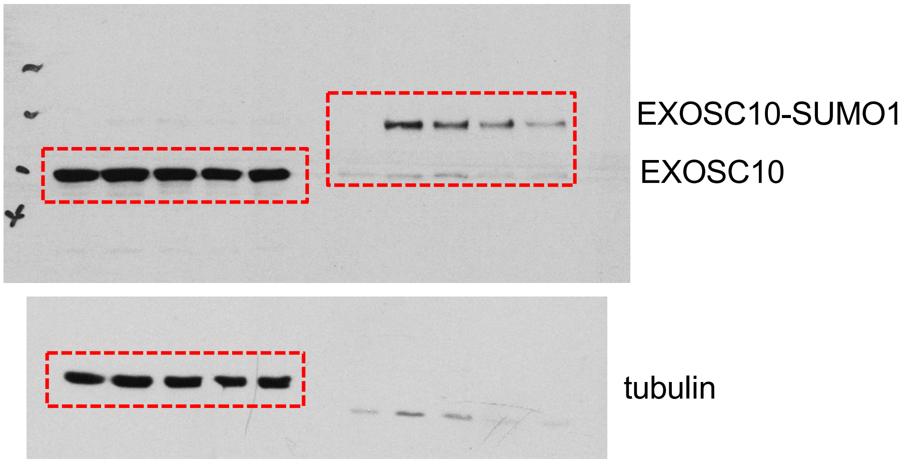

Figure S7B

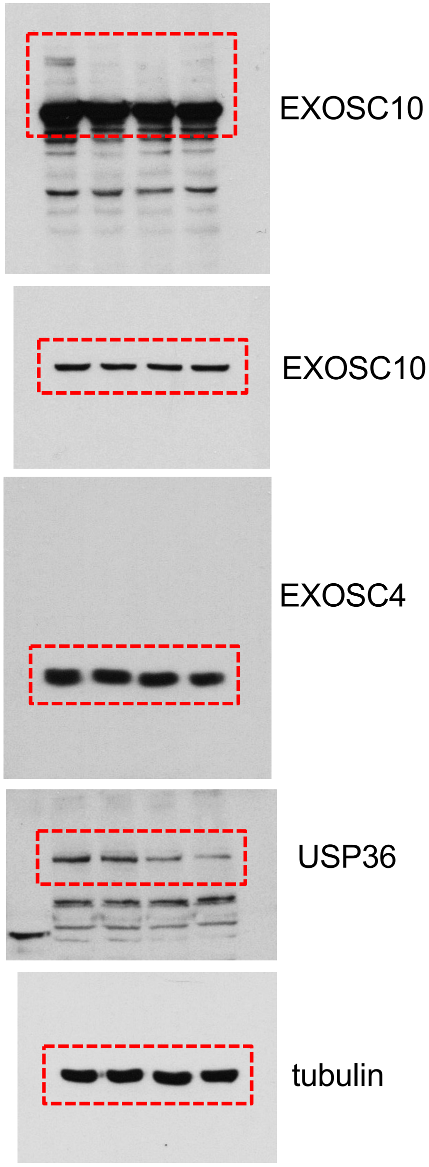

Figure S7C

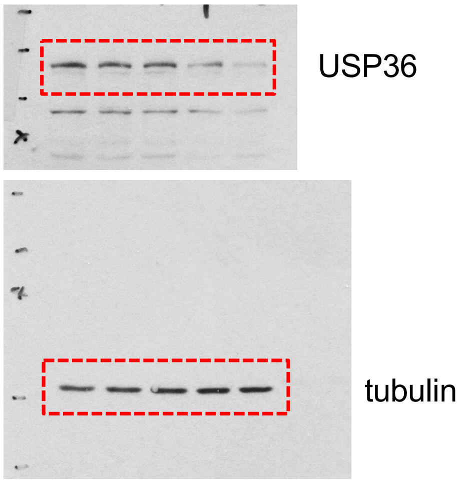

Supplement: gkad140_Supplemental_Files [file gkad140_supplemental_files.zip › uncropped data for sup Figs.pdf]
